# Type I CRISPR-Cas provides robust immunity but incomplete attenuation of phage-induced cellular stress

**DOI:** 10.1093/nar/gkab1210

**Published:** 2021-12-20

**Authors:** Lucia M Malone, Hannah G Hampton, Xochitl C Morgan, Peter C Fineran

**Affiliations:** Department of Microbiology and Immunology, University of Otago, PO Box 56, Dunedin 9054, New Zealand; Department of Microbiology and Immunology, University of Otago, PO Box 56, Dunedin 9054, New Zealand; Department of Microbiology and Immunology, University of Otago, PO Box 56, Dunedin 9054, New Zealand; Department of Microbiology and Immunology, University of Otago, PO Box 56, Dunedin 9054, New Zealand

## Abstract

During infection, phages manipulate bacteria to redirect metabolism towards viral proliferation. To counteract phages, some bacteria employ CRISPR-Cas systems that provide adaptive immunity. While CRISPR-Cas mechanisms have been studied extensively, their effects on both the phage and the host during phage infection remains poorly understood. Here, we analysed the infection of *Serratia* by a siphovirus (JS26) and the transcriptomic response with, or without type I-E or I-F CRISPR-Cas immunity. In non-immune *Serratia*, phage infection altered bacterial metabolism by upregulating anaerobic respiration and amino acid biosynthesis genes, while flagella production was suppressed. Furthermore, phage proliferation required a late-expressed viral Cas4 homologue, which did not influence CRISPR adaptation. While type I-E and I-F immunity provided robust defence against phage infection, phage development still impacted the bacterial host. Moreover, DNA repair and SOS response pathways were upregulated during type I immunity. We also discovered that the type I-F system is controlled by a positive autoregulatory feedback loop that is activated upon phage targeting during type I-F immunity, leading to a controlled anti-phage response. Overall, our results provide new insight into phage-host dynamics and the impact of CRISPR immunity within the infected cell.

## INTRODUCTION

Phages are ubiquitous and play a fundamental role modulating the turnover of bacterial communities in all environments (1,2). As obligate parasites, phages orchestrate the temporal expression of their genes to ensure the correct progression of infection, with infection usually divided into early, mid and late stages (3). Most early phage genes encode short uncharacterized proteins involved in redirecting host transcriptional machinery and metabolism towards phage replication (4). Additionally, these early genes, are often involved in blocking bacterial defence systems and include anti-CRISPRs (5–9). In contrast to the early expressed genes, those expressed during mid infection are required for DNA replication and nucleotide biosynthesis, whilst gene products from late infection are involved in the synthesis of new viral particles, DNA packaging and host lysis (10,11).

Bacteria encode an arsenal of defence mechanisms to prevent phage infection, such as restriction modification (R-M) systems (12), abortive infection (Abi) systems that induce altruistic ‘suicide’ of infected cells (13), and CRISPR-Cas systems (14,15). CRISPR-Cas systems provide adaptive immunity and are divided into two classes (I and II), several types (I-VI) and subtypes (16). Type I CRISPR-Cas systems are the most widespread in bacteria and archaea (17) and possess CRISPR associated (Cas) proteins that are typically encoded proximal to one or more CRISPR arrays. These arrays harbour short sequences derived from foreign genetic elements (spacers) interspaced by repeats. CRISPR-Cas immunity is achieved through: (i) adaptation, (ii) expression and processing, and (iii) interference (18). In type I CRISPR-Cas systems, the Cas complex recognises targets complementary to the crRNA, leading to recruitment of Cas3 which cleaves DNA and arrests phage infection (19–21). Bacteria often harbour multiple CRISPR-Cas systems, among other bacterial immune systems. For example, *Serratia* sp. ATCC 39006 (*Serratia*), an environmental Gram negative bacterium, encodes three CRISPR-Cas systems (type I-E, I-F and III-A) (22–24), one R-M system and 32 toxin–antitoxin (TA) systems (25).

Recent studies have led to a deeper understanding of the fate of infected cells during CRISPR immunity. RNA targeting by type III and VI CRISPR-Cas systems have been suggested to elicit protection through growth arrest (26) or death of the infected cell (27–29). In contrast, DNA targeting CRISPR-Cas systems have been presumed to provide strong protection through foreign DNA degradation, without disturbing other cellular pathways (13). However, in *Pectobacterium*, the outcome of infected cells during type I-F CRISPR-Cas targeting varies with the nature of the mobile genetic element (30). While plasmids are cleared and result in cell survival, infection with virulent phages leads to abortive infection, suggesting the path to CRISPR immunity is more complex than previously thought. Similarly, type I-E protection varies with the type of infection strategy employed by the phage (31). A recent study revealed that phage genes can be transcribed throughout the infection period even in the presence of robust CRISPR protection (32). Yet, the impact of CRISPR interference in the infected cell and its effect on phage-host dynamics remains poorly understood. Here, to understand the interplay between CRISPR-Cas immunity and phage development we evaluated the phage and host transcriptional response during infection in the presence or absence of immunity.

## MATERIALS AND METHODS

### Culture conditions and phage lysate preparation

*Serratia* sp. ATCC 39006 (*Serratia*) and *Escherichia coli* ST18 were grown overnight in lysogeny (LB) broth at 30 and 37°C, respectively in shaking conditions (160 rpm). When grown on plates, LB with agar (LBA, 1.5% w/v) was used and plates were incubated at an appropriate temperature until colony formation. When needed, antibiotics and supplements were added to the LB and LBA: Chloramphenicol (Cm; 25 μg/ml), Kanamycin (Km; 50 μg/ml), Tetracycline (Tc; 10 μg/ml), Gentamycin (Gm; 30 μg/ml) and 5-aminolevulinic acid (ALA; 50 μg/ml). New phage stocks were prepared as described elsewhere (23) using the double agar overlay method. Briefly, 100 μl of bacterial overnight culture and 100 μl of phage lysate were added to 4 ml of molten LBA overlay (0.35% w/v) and poured onto LBA plates. The phage concentration used was enough to produce almost confluent lysis. After an overnight incubation at 30°C, the overlays were pooled into a collection tube. Two drops of chloroform (NaHCO_3_-saturated) were added and the sample was vortexed to lyse the cells. Finally, cellular debris was removed by centrifugation (2000 *g* for 20 min) and the phage stock was transferred into a new tube and stored at 4°C. To calculate the phage titre, 10-fold serial dilutions were made in phage buffer (10 mM Tris Base (pH 7.4), 10 mM MgSO_4_, 0.01% w/v gelatine) and spotted (20 μl) onto LBA overlay previously seeded with 100 μl of *Serratia*. After an overnight incubation at 30°C, plaques were counted and phage titre was expressed as plaque forming units (pfu)/ml.

### Primers, plasmids and strains

Primers, plasmids and strains used in this study are detailed in Supplementary Tables S1, S2 and S3, respectively.

### Phage isolation

Phage JS26 was isolated from sewage samples collected from the Tahuna Waste Water Treatment Plant in Dunedin, New Zealand (45°54'16.1‘S; 170°31'16.8’E). Enrichment for *Serratia* phages was performed by inoculating an overnight *Serratia* culture with 100 μl of sewage sample and incubating overnight at 30°C under shaking conditions (160 rpm). A 10-fold dilution series was prepared and plated onto a top agar overlay seeded with *Serratia* overnight culture. Plaques showing different morphologies were picked and used to infect a new *Serratia* culture. This step was repeated until homogenous looking plaques were obtained to ensure phage purity.

### Genome sequencing, annotation and comparative genomics

Phage genomic DNA (gDNA) was extracted from a high titre phage stock (∼10^9^ pfu/ml) using the cetyltrimethylammonium bromide (CTAB) method described elsewhere (33). Samples were cleaned using the DNeasy Blood & Tissue Kit (QIAGEN) following the manufacturer's instructions and DNA was quantified using the Qubit dsDNA HS Assay Kit and the Qubit Fluorometer (2.0) (invitrogen) following the manufacturer's instructions. Isolated gDNA was sent to the Massey Genome Service (New Zealand) where libraries were prepared using the Nextera XT DNA Library Preparation Kit (Illumina), QC was checked with the Quant-iT dsDNA HS Assay for quantification and analysed using SolexaQA++, fastQC and fastQsceen. Sequencing was performed using Illumina MiSeq (2 × 150 bp) and the resulting reads were processed and trimmed using SolexaQA++ (v3.1.7.1). The genome was assembled using SPAdes 3.9 (34), annotated with RASTtk (2.0) (35) and manually curated using BLASTp (2.10.0) (Supplementary Table S5). To identify putative tRNAs, tRNAscan-SE v. 2.0 (36) was used. The final genome sequence was deposited in GenBank under the accession number MN505213.1 and visualized using EasyFig (2.2.2) (37). Related phages were identified using PAirwise Sequence Comparison (PASC) (38), the phylogenetic tree was built through whole genome blast using VIrus Classification and Tree building Online Resource (VICTOR) (39) using distance formula *d*_6_ and branch support was inferred from 100 pseudo-bootstrap replicates each and modified with FigTree (v1.4.3).

### Proteomics

A *Serratia* culture (25 ml) was mixed with 500 μl of high titre phage stock (∼10^9^ pfu/ml) and grown overnight at 30°C in shaking conditions (160 rpm). Cell debris were removed by centrifugation (2000 *g* for 20 min) and the supernatant containing phages was concentrated and purified with a sucrose cushion. Briefly, phage samples were pipetted onto a 20% (w/v) sucrose solution underlay. The sample was separated by centrifugation (120 000 *g* for 4 h at 20°C) and the pellet was resuspended in 250 μl of no-gelatin phage buffer and dialysed overnight against 2 l of milliQ water at 4°C. The phage solution was pooled together and cleaned from cell debris by centrifugation (10 000 *g* for 20 min). Next, a CsCl gradient was prepared by adding 2 ml of 1.2 g/ml and 1.6 g/ml CsCl solutions into a centrifuge tube. The sample was separated by centrifugation (120 000 *g* for 4 h at 20°C), and the white phage interface that formed in the CsCl gradient was harvested using a Pasteur pipette. The concentrated phage stock was washed by adding no-gelatin phage buffer and pelleted by an ultracentrifuge step (120 000 *g* for 2 h at 20°C). The pellet was resuspended in 100 μl milliQ water and stored at 4°C. For the proteomic analysis, 75 μl of phage sample was mixed with 20 μl of 1/10 (v/v) β-mercaptoethanol 4× SDS-buffer and incubated for 5 min at 95°C. The sample was separated by electrophoresis in a 12% (w/v) SDS-polyacrylamide gel for 1 h at 150 V. Page ladder (Thermo Fisher Scientific) was used as a reference. The gel was fixed for 2 h in 10% (v/v) acetic acid, 40% v/v ethanol. Finally, the gel was stained overnight in four parts of Colloidal Coomasie Blue stain (0.1% G250 w/v, 10% w/v ammonium sulphate and 2% v/v *ortho*-phospholic acid) and one part methanol.

Proteomic analysis of the phage structural proteins was performed as described elsewhere (40). Briefly, the gel lane was subjected to in-gel digestion with trypsin and analysed by protein identification by liquid chromatography–coupled tandem mass spectrometry (LC–MS/MS) in the Centre for Protein Research (University of Otago). Peptide reconstruction was performed with an Ultimate 3000 nano-flow uHPLC-System (Dionex Co., Thermo Fisher Scientific; Waltham, MA, USA) in-line coupled to the nanospray source of an LTQ-Orbitrap XL mass spectrometer (Thermo Scientific; Waltham, USA). Raw spectra were processed through the Proteome Discoverer software (Thermo Fisher Scientific, Waltham, MA, USA) using default settings to generate peak lists. Peak lists were then searched against a combined amino acid sequence database containing all JS26 sequence entries (GenBank accession number MN505213.1, 84 entries) integrated into the full SwissProt/UniProt sequence database using the Sequest HT (Thermo Fisher Scientific), Mascot (www.matrixscience.com) and MS Amanda search engines (Supplementary Table S6).

### Electron microscopy

To examine phage by transmission electron microscopy (TEM), 10 μl of high titre phage stock (∼10^9^ pfu/ml) was loaded onto plasma-glow discharged carbon coated 300 mesh copper grids. After 60 s, the excess specimen was removed by blotting and 10 μl of 1% (w/v) phosphotungstic acid (PTA) (pH 7.2) was applied to the grid to stain the samples and blotted off immediately. The grids were analyzed in the Otago Micro and Nano Imaging (OMNI) facility and viewed in a Philips CM100 BioTWIN transmission electron microscope (Philips/FEI Corporation, Eindhoven, Holland) and images captured using a MegaView lll digital camera (Soft Imaging System GmbH, Münster, Germany).

### Efficiency of plaquing assay

To assess phage infectivity in different *Serratia* strains, an efficiency of plaquing (EOP) assay was performed. Serial 10-fold dilutions of high titre phage stocks (∼10^9^ pfu/ml) were spotted onto LBA overlays seeded with *Serratia* (100 μl). After the spots were dry, the plates were incubated at 30°C overnight. The EOP was calculated as the ratio between the pfu/ml in *Serratia* strains and the wild-type control. All conditions were repeated in biological triplicates and the data was plotted as the mean ± SD.

### Phage infection time courses

*Serratia* strains were grown to exponential phase (OD_600_ = 0.3) and diluted to an OD_600_ = 0.05. Bacterial cultures (180 μl) were pipetted into 96-well plates and infected with 20 μl phage lysate to produce a multiplicity of infection (moi) = 0, 1 and 10. The plates were incubated in a Varioskan Flash plate reader (Thermo Fisher Scientific) for 20 h at 30°C and shaking (240 rpm) and OD_600_ measurements were taken every 12 min. The experiment was repeated in biological triplicates and the data was plotted as the mean ± SD.

### Phage receptor characterization

Receptor identification was performed by transposon mutagenesis. Overnight cultures of donor (*E. coli* ST18 pKRCPN2 carrying transposon Tn-DS1028*uidA*Km) and recipient (*Serratia*) strains were adjusted to an OD_600_ = 1, washed free from antibiotics and mixed in equal ratios. The samples were spotted (20 μl) onto LBA + ALA and incubated overnight at 30°C to allow conjugation. Next, the spots were scraped and resuspended in LB + Km to select for the transposon insertion events. The mutant pool was grown overnight and seeded onto an LBA overlay. High titre JS26 stocks (∼10^9^ pfu/ml) were spotted (20 μl) onto the lawn and plates were incubated at 30°C until colonies appeared within the phage spot. Phage resistant mutants were restreaked onto a new plate to ensure purity.

Transposon insertion sites were identified by arbitrary PCR (41). A first round of random colony PCR was performed on phage resistant clones using a random primers PF106, PF107 and PF108 and transposon nested primers PF226 and PF1212. PCR products were cleaned using the GFX™ PCR DNA and Gel Band Purification Kit (GE Healthcare) and used as DNA template for a second round of PCR with adapter primer PF109 (that binds to the 5′ ends of PF106-PF108) and Tn-DS1028*uidA*Km nested primers (either PF226 or PF1212). Bands were extracted, sequenced and mapped against *Serratia* genome to detect the transposon insertion site. EOP assay were performed on transposon mutants (PCF619, PCF620, PCF621, PCF623) to test phage resistance with an Δ*flhDC* mutant (PCF879) used as a control. Moreover, the swimming ability of the mutants was evaluated in a motility assay. The mutants were stabbed onto LBA (0.35% w/v) and grown overnight at 30°C. Motility was assessed by measuring the halo of swimming from the inoculated spot and compared with a *Serratia* WT as the control.

### One step growth curve

A *Serratia* culture (25 ml) was grown up to exponential phase (OD_600_ = 0.3) and infected with JS26 at an moi = 0.1. The culture was incubated at 30°C in shaking conditions (160 rpm) and samples were taken at different time point (5, 20, 40, 60, 80, 100, 120 140, 160 min post infection (mpi)). At each time point, a 100 μl aliquot was taken and mixed with 900 μl of LB. The samples were immediately diluted in a 10-fold series and spotted (20 μl) onto LBA overlays previously seeded with *Serratia*. The experiment was repeated in three biological replicates and the data was plotted as mean of pfu/ml ± SD.

### Cell survival assay

To determine the moi at which synchronicity of infection was achieved, a cell survival assay was performed. *Serratia* cultures (5 ml) were grown up to an early exponential phase (OD_600_ = 0.3) and infected with a range of moi (moi = 0, 0.1, 1, 25, 50). The infected cultures were incubated at 30°C, shaking (160 rpm) and samples were taken (500 μl) at time points 0, 5 and 25 mpi. The aliquots were mixed with 500 μl LB and centrifuged (17 000 *g* for 2 min) to remove free phages. The bacterial pellet was washed with 1 ml of LB, resuspended in 500 μl LB and used to prepare in 10-fold serial dilutions. The bacterial dilutions were spotted (20 μl) onto LB plates. The percentage of cell survival was calculated as cfu/ml in infected samples/cfu/ml in uninfected control × 100%. The experiment was repeated in biological triplicates and the data was plotted as mean ± SD.

### Generation of native type I-E and I-F anti-phage strains

*Serratia* strains harbouring anti-JS26 spacers (Supplementary Tables S3 and S4) in their type I-E and I-F chromosomal arrays (CRISPR1 and CRISPR2 respectively) were generated by primed spacer acquisition (42). Plasmids pPF1257 (type I-E priming vector) and pPF1258 (type I-F priming vector), carrying a phage fragment and a protospacer primed by a spacer in CRISPR1 and CRISPR2, respectively, were constructed as follows. A fragment (∼1 kb) of the tape measure gene (*gp61*) was amplified by PCR (using primers PF2296 and PF2297) from phage gDNA and digested with SpeI and KpnI. The insert was cloned into the two priming vectors (pPF1125 and pPF1126) previously digested by the same enzymes. Plasmids were transformed into *E. coli* ST18, plated onto LBA + ALA + Cm and cloning was checked by PCR using primers PF1403 and PF1372, and confirmed by sequencing. The resulting plasmid was conjugated into *Serratia* by mixing equal ratios of donor and recipient strains and spotting mating spots onto LBA + ALA and incubated at 30°C overnight. The mating spots were streaked onto LBA + Cm to select for transconjugants.

Colonies were grown overnight in LB in the absence of antibiotic selection to allow for plasmid targeting and cultures passaged overnight for 2–3 days. Aliquots were taken each day, and dilutions were plated onto LBA and LBA + Cm to check for plasmid loss. Array expansion was checked by PCR screening with PF1989/PF1887 (CRISPR1) and PF1990/PF1888 (CRISPR2), and acquisition of anti-JS26 spacers was confirmed by sequencing.

### RNA extraction and sample preparation and RNA-seq analysis

*Serratia* WT or anti-JS26 strains (PCF524 and PCF525) were grown to exponential phase (OD_600_ = 0.3) in 25 ml LB at 30°C and in shaking conditions (160 rpm). Cultures were infected at an moi = 50 and 2 ml samples were taken at 0 (prior to phage infection), 5, 20 and 40 mpi. Immediately after extraction, the samples were centrifuged (17 000 *g* for 1 min at 4°C) to remove the free phage and the bacterial pellet was resuspended in 2 ml of RNAlater (Invitrogen) and stored at –20°C. The experiment was repeated in biological triplicates.

Total RNA was extracted using the RNeasy kit (QIAGEN). Briefly, samples resuspended in RNAlater were harvested by centrifugation (17 000 *g* for 1 min at 4°C) and resuspended in 250 μl of 10 mM Tris–HCl (pH 8.0). The resuspended pellet was transferred to a pre-chilled bead beater tube containing 350 μl of RLT buffer (lysis buffer) and β-mercaptoethanol. Cell lysis was performed with a bead beater, using three 10 s pulses interspaced by a 1 min incubation in ice. The samples were chilled for 3 min on ice and cellular debris was removed by centrifugation (17 000 *g* for 1 min at 4°C). The homogenate was transferred into a clean Eppendorf and 1.5× volume of 100% ethanol was added and mixed by vortexing. The sample was transferred into the RNeasy mini spin column, centrifuged (>8000 *g* for 15 s) and the flow through discarded. Next, the column was washed twice with 500 μl of Buffer RPE (washing buffer) followed by centrifugation (>8000 *g* for 15 s). RNA was eluted by adding 50 μl of RNase free water followed by centrifugation (>8000 *g* for 1 min). The sample was treated with TURBO DNase (Invitrogen) following the manufacturer's instructions. Finally, impurities were removed by centrifugation (>8000 *g* for 1 min) and the RNA samples were transferred to a new tube and stored at –20°C.

The absence of DNA contamination was confirmed by PCR using primers PF796 and PF797 that amplify a fragment encoding the *flhDC* genes in *Serratia*. RNA concentration was estimated using a nanodrop and the quality assessed by an RNA nano chip on the Bioanalyzer (Agilent). The total RNA samples were ribo-depleted using Ribo-zero rRNA removal kit (epicentre) and directional cDNA libraries were prepared with the TruSeq stranded mRNA library preparation kit (Illumina) following manufacturer's instructions.

The samples were sequenced in the Otago Genomic Facility (OGF, New Zealand) using Illumina HiSeq 2500 v4 Rapid single-read sequencing, with ∼10 million reads (101 bp) generated per sample. The reads were trimmed using Trimmomatic (43) and their quality evaluated through FASTQC analysis. Finally, raw sequence reads were aligned to *Serratia* (accession number CP025085) and JS26 (accession number MN505213.1) genomes using Bowtie2 with default parameters (44). The alignment was converted to BAM files format using SAMtools (45). The differential expression between the uninfected controls (timepoint 0) and the infected samples (5, 20 and 40 mpi) was evaluated using the DESeq2 (46) R/Bioconductor package (3.5.2) with a Wald test, followed by a Benjamini and Hochberg procedure. All sets were defined by a false discovery rate of 10%. Genes differentially expressed in *Serratia* WT and anti-JS26 I-E and I-F strains are listed in Supplementary Tables S7, S8 and S9, respectively. Classification of differentially expressed genes was performed using the gene set enrichment analysis platform (GSEA-pro V3.0) using operon, Gene Ontology (GO) (47) and the Kyoto Encyclopedia of Genes and Genomes (KEGG) (48) databases.

### Adaptation assay

To determine if the phage-encoded *cas4* homologue affected spacer acquisition in *Serratia*, *cas4* was cloned into an overexpression vector under an arabinose inducible promoter (with *cas4*, pPF2291; empty vector, pPF783) and overexpressed during adaptation assays. The *cas4* gene was amplified from JS26 gDNA through PCR using primers PF4692 and PF4693. Gibson assembly (NEBuilder HiFi DNA Assembly) was used to clone the insert into the expression vector pPF783 previously digested with restriction enzymes EcoRI and SphI. The construct was transformed into *E. coli* ST18 and conjugated into *Serratia*. Adaptation assays during *cas4* overexpression were performed as previously described (22). Briefly, plasmids pPF719 and pPF953 (non-targeted ‘naïve’ control), pPF1233 and pPF1048 (high and medium ‘primed’ type I-E) and pPF1242 and pPF1243 (high and medium ‘primed’ type I-F) were conjugated from *E. coli* ST18 into *Serratia* carrying pPF783 or pPF2291. Transconjugants were streaked onto LBA with Tc and Gm to ensure maintenance of the two plasmids. Single colonies were used to inoculate 5 ml LB cultures supplemented with arabinose (0.05% w/v) and Gm. These were incubated overnight at 30°C under shaking condition (160 rpm) and passaged for 3 days by daily transfer of 10 μl of culture into 5 ml of fresh LB supplemented with arabinose (0.05% w/v) and Gm. CRISPR array expansion was determined by PCR directly on cells from passaged cultures using primers PF633 and PF2177 for the type I-E array (CRISPR1) and PF1888 and PF1990 for the type I-F array (CRISPR2). PCR products were run on 3% agarose gels with a 1 kb + DNA ladder (invitrogen), stained with ethidium bromide and visualized under UV light. The experiment was performed in biological triplicates.

### CRISPRi of the phage *cas4* homologue

To determine if the JS26 *cas4* homologue was required for phage replication, EOP and phage infection time courses were performed on *Serratia* carrying a dCas9 expression vector with (pPF2786) or without (pPF1755) a single guide RNA (sgRNA) targeting the *cas4* homologue in JS26. EOP and phage infection time courses experiments were performed as previously described with the addition of arabinose (0.1% w/v) to induce expression of dCas9 and Km for plasmid maintenance.

## RESULTS

### JS26 is a siphovirus that infects *Serratia* in a flagellum-dependant manner

**Figure 1.**
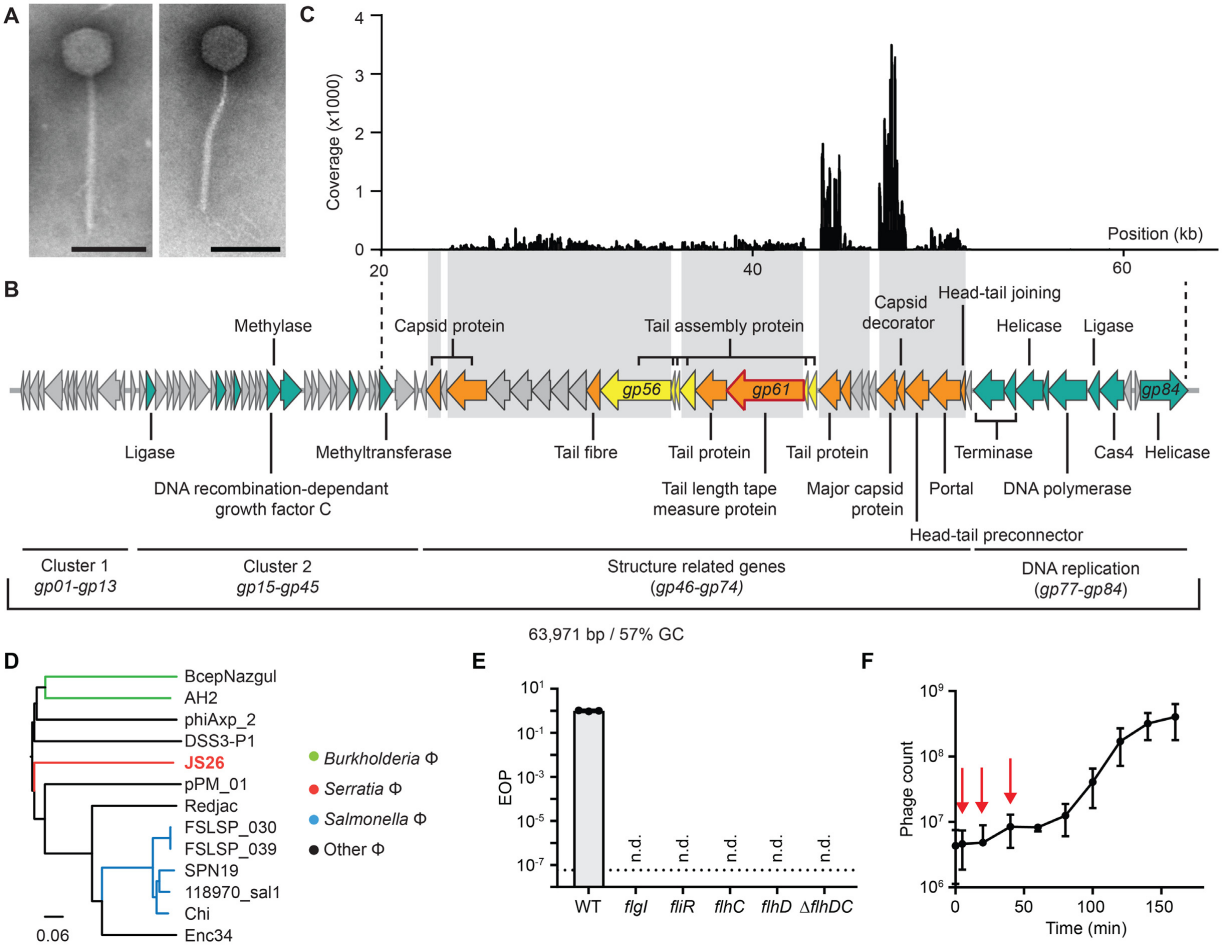
JS26 is a flagellatropic siphovirus that infects *Serratia*. (**A**) Electron micrographs of JS26 particles (scale bars: 100 nm). (**B**) Phage genome. Gene classification: nucleic acid manipulation (green), structural (orange), structure assembly (yellow) and hypothetical (grey) (see Supplementary Table S5). Structural proteins confirmed by MS are highlighted in grey boxes. (**C**) Peptide coverage from structural proteomic analysis (see Supplementary Table S6). (**D**) Genome BLAST Distance Phylogeny tree of JS26 and related phages: *Burkholderia* phages (BcepNazgul and AH2), *Salmonella* phages (FSLSP_030, FSLSP_039, SPN19, 118970_sal1, Chi), *Achromobacter* phage (phiAxp_2), *Ruegeria* phage (DSS3-P1), *Proteus* phage (pPM_01), *Providencia* phage (Redjac) and *Enterobacter* phage (Enc34). Branch lengths are scaled according to the distance formula (*d6*) (39). (**E**) Efficiency of plaquing (EOP) assay on receptor mutant stains: *flgI* (PCF619), *fliR* (PCF621), *flhC* (PCF623) and *flhD* (PCF620) and chromosomal knock-out Δ*flhDC* (PCF879). Not detected (n.d.) was noted when no plaque formation was observed at the limit of detection (dotted line). (**F**) One-step growth curve (pfu/ml) of JS26 at moi = 0.01. Red arrows indicate early, middle and late infection at which samples were taken to perform RNA-sequencing. In (E) and (F), data is represented as mean of three biological replicates ± SD.

To broaden our knowledge about phage-host transcriptional responses and the effects of CRISPR-Cas immunity, CRISPR-Cas sensitive phages are required. Since the phage that we previously isolated evades CRISPR-Cas immunity (23), a new *Serratia* phage (JS26) was isolated from a sewage sample in New Zealand and characterized. Electron microscopy revealed that the phage was a member of the *Siphoviridae* family with an isomeric icosahedral capsid (∼70 nm diameter) and a flexible tail (∼210 nm) (Figure 1A). The phage was sequenced and assembled, revealing a dsDNA genome of 63 971 bp with a GC content of 57%, which is higher than the GC content of its *Serratia* host (47%). In total, 84 open reading frames (ORFs) were predicted, of which 50 were annotated as hypothetical or conserved phage proteins of unknown function (Figure 1B, Supplementary Table S5). The genome of JS26 contains no predicted tRNAs and is organised into clusters of genes with similar function. Cluster 1 contains short hypothetical genes of unknown function (*gp01*-*gp13*). Genes encoded between *gp15*-*gp45* (cluster 2) are involved in DNA manipulation (ligase, *gp16*; methylases *gp32* and *gp39*; DNA-recombination-dependant growth factor C, *gp33*). Structural genes (*gp46–gp74*) and viral assembly chaperones, cluster together with genes involved in DNA packaging (portal, *gp72* and terminase, *gp75–76*, cluster 3). Finally, genes encoded at the terminal end of the genome (cluster 4) are predicted to be involved in DNA replication (*gp77–gp84*) among which, a putative *cas4* homologue was identified (*gp81*). Cas4 is a nuclease present in some CRISPR-Cas systems and is involved in processing and acquisition of spacers during adaptation (49), yet homologues of *cas4* are widespread in different mobile genetic elements and are found in transposable elements (50), archaeal viruses (51) and phages (52). No predicted lysis genes with similarity to characterized lysis genes were identified.

To investigate the phage structural composition, a proteomic analysis was performed on purified mature phage virions (Figure 1C and Supplementary Table S6). From the 21 proteins confirmed by mass spectrometry, 11 corresponded to the putative structural genes and the rest were hypothetical proteins encoded in the structural gene cluster. Peptide mapping revealed that the major capsid protein (Gp69) is the most abundant, followed by a tail protein (Gp64) (Figure 1C). Next, a phylogenetic analysis was performed to identify closely related phages. We determined there are no close relatives to JS26 (<35% identity at DNA level), yet this phage is distantly related to other siphoviruses that infect a wide range of bacterial species from the Phylum Proteobacteria (Figure 1D).

To begin to define the phage-host interaction, we identified the receptor for JS26 on *Serratia*. We performed a random Tn5 transposon mutagenesis and selected for resistance to JS26. Mutants carrying transposon insertions in genes involved in flagella biosynthesis (*fliR*), structure (*flgI*) and regulation (*flhD* and *flhC*), were resistant to JS26 infection (Figure 1E). The same phenotype was observed with an Δ*flhDC* mutant, a strain deficient in the master flagella regulator (Figure 1E). To characterize the dynamics of JS26 infection, a one-step growth curve was performed, which revealed that infection by JS26 has a latent period of 60 min, after which viral progeny are released with a burst size of ∼140 pfu/infected cell (Figure 1F). Overall, these results show that JS26 is a unique siphovirus that infects *Serratia* in a flagellum-dependant manner.

### Genes in JS26 are temporally regulated

**Figure 2.**
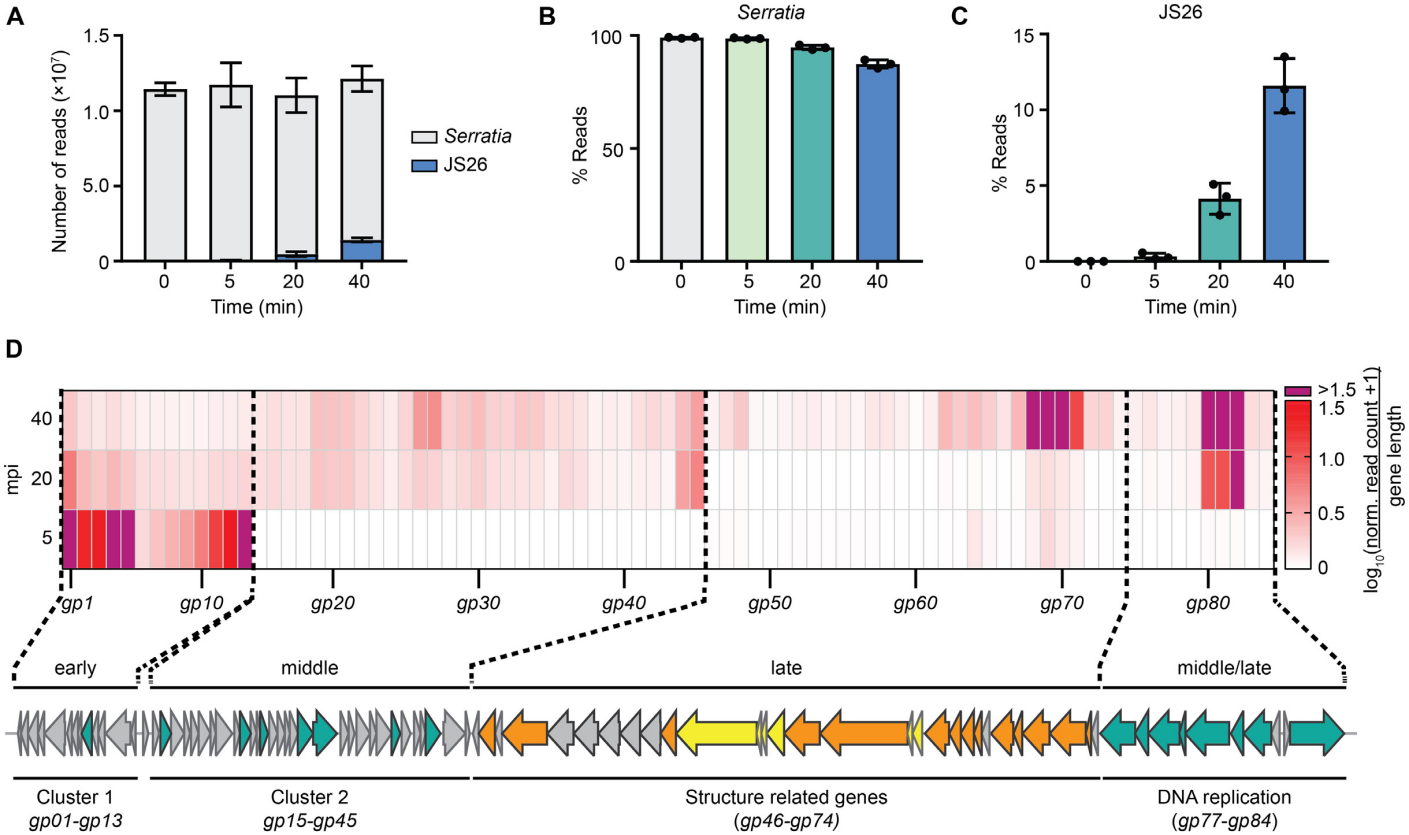
JS26 temporal gene expression profile. (**A**) Total number of reads mapping to *Serratia* (light grey) and phage JS26 (blue). Percentage (%) of reads mapping to (**B**) *Serratia* and (**C**) JS26 throughout infection. In (A–C), data are represented as the mean of biological triplicates ± SD. (**D**) Temporal analysis of phage gene expression. Changes in gene expression were represented as the mean of the normalized read counts of each timepoint against the uninfected control (DESeq2 normalization) divided by gene length (bp) and shown as log_10_(*x* + 1). Values for highly expressed genes that were above the defined range are indicated in purple. *gp14* was omitted from the diagram as no reads mapped to this predicted gene. For raw read density across phage genome see Supplementary Figure S1A–D.

To investigate phage infection and its effect on the host, *Serratia* was infected with JS26 and we analysed the transcriptional response by strand-specific RNA sequencing. To assess temporal changes in phage and host transcript abundance, samples were taken before infection (0 minutes post infection (mpi), uninfected control) and at early (5 mpi), middle (20 mpi) and late (40 mpi) stages of infection, before cell burst and phage release occurred (Figure 1F). A high multiplicity of infection (moi = 50) was used to maximise synchronicity of infection. RNA sequencing reads (∼10^7^ reads per sample) were aligned to either *Serratia* or JS26 genomes (Figure 2A) and changes in bacterial and phage gene expression were evaluated by comparing read abundance in each time point against the uninfected control. Throughout infection, most reads mapped to the host, with more than 85% of reads corresponding to *Serratia* at the late stage of infection (Figure 2B). However, phage read abundance increased as infection progressed, accounting for ∼2%, ∼4% and ∼11% of total reads in early, middle and late stage of infection, respectively (Figure 2C).

To study the phage transcriptional profile, reads were mapped back to JS26 and gene expression levels were evaluated (Figure 2D). The analysis of gene expression revealed that gene clusters are temporally expressed (Figure 2D). Cluster 1, which is mostly composed of hypothetical genes of unknown function, is expressed early in infection (Figure 1B). Consistent with other phage studies (11), genes expressed during mid-infection (Cluster 2 and 4) are mostly involved in DNA metabolism and replication. As expected, late genes are involved in virion assembly, DNA packaging and replication (Cluster 3). Notably, the gene cluster including a ligase (*gp80*), a *cas4* homologue (*gp81*) and a hypothetical gene (*gp82*) are strongly expressed throughout middle and late stages of infection. Expression of these genes is considerably higher than other genes, suggesting an essential role during phage development (Figure 2D & Supplementary Figure S1A–D). Solo *cas4* encoded by MGEs have been suggested to have an anti-defence role (53) and a study showed that overexpression of *cas4* from archaeal virus SSVRH leads to a reduction in spacer acquisition in the type I-A CRISPR-Cas system in *Sulfolobus islandicus* (54). In contrast, a separate study suggested that the endonuclease activity of the Cas4 homologue encoded by archaeal virus SIRV2 could be involved in viral genome processing during phage DNA replication (51). To investigate if the *cas4* homologue encoded by JS26 had a role in adaptation, we overexpressed *cas4* from a plasmid and evaluated array expansion during naïve and primed adaptation (Supplementary Figure S1E). No difference in type I-E and type I-F CRISPR adaptation was observed, suggesting that the phage encoded *cas4* homologue does not influence spacer acquisition. To evaluate whether the phage encoded *cas4* homologue has a role in phage replication, we silenced its expression with CRISPR-dCas9 during infection. Silencing *cas4* led to a reduced efficiency of plaquing (Supplementary Figure S1F) and protected bacteria from phage during infection in liquid culture (Supplementary Figure S1G). The *cas4* knockdown might also have a polar effect on the genes downstream in the operon, which are involved in DNA replication. Given its genetic context, our results suggest that Cas4 and/or the genes encoded in the vicinity are necessary to produce a successful infection in the absence of CRISPR-Cas immunity.

Overall, our results demonstrate that functional modules of phage JS26 genes are temporally expressed; while genes expressed during early infection encode mostly short proteins of unknown function, genes expressed in mid-infection are involved in DNA replication and late genes are related to structure and phage production. Here, we show that the *cas4* homologue encoded by JS26 is probably involved in phage replication, rather than an anti-CRISPR strategy.

### The host response is divided into an early and mid-late response

**Figure 3.**
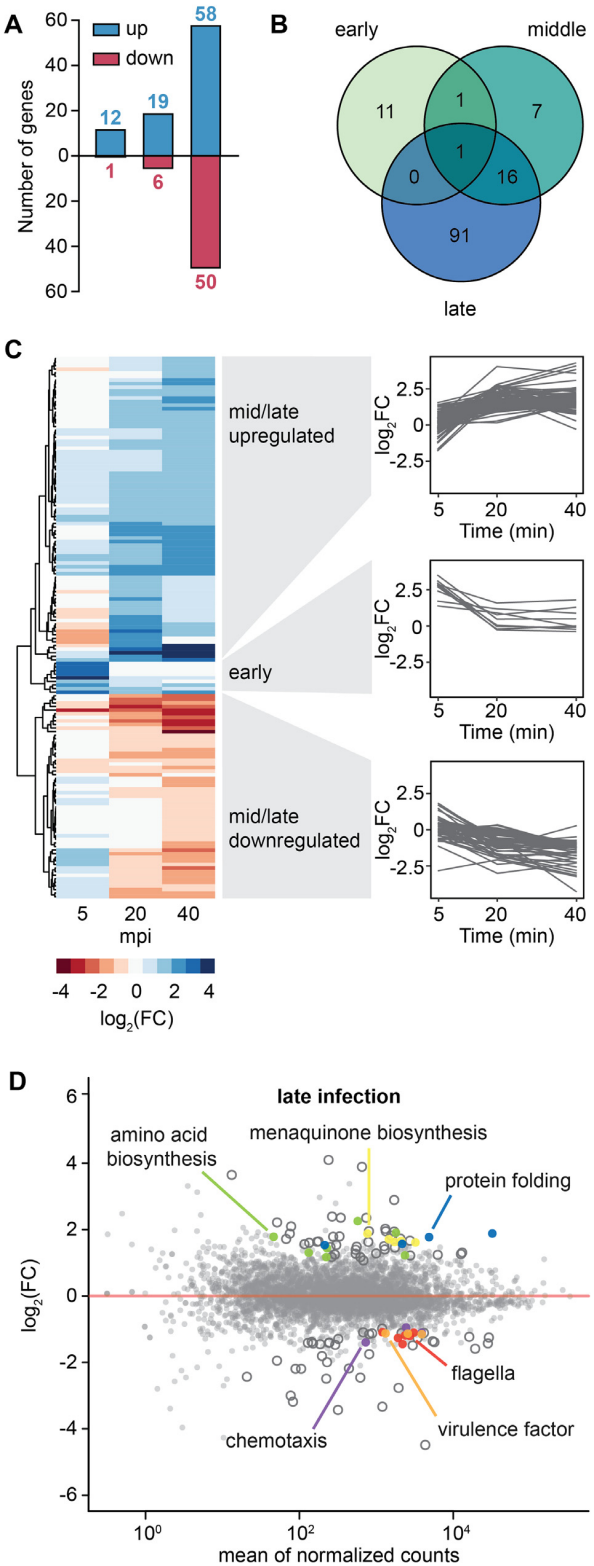
The host response is mostly altered at late stages of infection. (**A**) Number of genes significantly up (blue) and down (red) regulated in *Serratia* throughout infection. (**B**) Venn diagram of genes significantly differentially expressed during early (5 mpi), mid (20 mpi) and late (40 mpi) infection. (**C**) Heat map of host genes significantly differentially expressed in early, middle or late infection. (**D**) Expression landscape in *Serratia* during late infection (40 mpi). Genes significantly differentially expressed (log_2_(FC) > 0.58 and *P*_adj_ < 0.05) are represented as open or coloured circles depending their function. *Serratia* genes differentially expressed during JS26 infection are listed in Supplementary Table S7.

During infection, the host transcriptional landscape can be altered by two factors: (i) phage hijacking of the host machinery to redirect metabolism towards virion production and (ii) the cell response to phage stress. To investigate the genome-wide impact of phage infection on *Serratia*, we compared changes in host transcript abundance throughout infection. As infection progressed, there was an increase in the number of host transcriptional changes with 13, 25 and 108 genes significantly differentially expressed (log_2_(FC) > 0.58 and *P*_adj_ < 0.05) in early, mid, and late stages of infection respectively (Figure 3A, B and Supplementary Table S7). The host transcriptional response to phage infection can be divided into two distinct steps: a minor unique initial reaction, followed by a prolonged mid-late response that was exacerbated during late infection (Figures 3B and 3C). In summary, our results demonstrate that during JS26 infection there is no major host reprogramming during early infection, and that most host transcriptional changes occur during mid and late infection.

### Genes involved in anaerobic respiration are upregulated during early stages of infection

At early infection, a few host transcriptomic changes occurred (12 and 1 genes significantly up and downregulated) (Figure 3A). Five minutes after phage infection, the nitrate reductase operon *narGHIJ* and the Ni/Fe-hydrogenase cytochrome β-subunit were upregulated and both are involved in anaerobic respiration (Supplementary Figure S2A) (55,56). During mid infection (20 mpi), 19 genes were significantly upregulated (Figure 3A), including those involved in glutathione biosynthesis – an antioxidant that protects against reactive oxygen species (57). Among the six genes significantly downregulated in mid infection were genes involved in carbon metabolism (pyruvate dehydrogenase complex transcriptional repressor *pdhR*, and pyruvate dehydrogenase *aceE*) (58) (Supplementary Figure S2B). The only gene upregulated throughout JS26 infection was *nhaA*, a Na^+^/H^+^ antiporter involved in the regulation of intracellular sodium, pH and essential for cell homeostasis (59) (Figure 3B). Overall, our results suggest that the initial host response involves a decrease in the general cell metabolism through the downregulation of carbon metabolism pathways and a switch to anaerobic respiration.

### Amino acid biosynthesis is upregulated while flagella are downregulated during late infection

Most transcriptional changes occurred during late infection, with 58 and 50 genes significantly up and downregulated, respectively (Figure 3A). As infection progressed, more genes involved in anaerobic respiration were upregulated, including the *menECBHDF* operon for menaquinone biosynthesis (Figure 3D). Menaquinone is important for anaerobic respiration as an electron carrier between membrane bound respiratory complexes (60). We also observed increased expression of genes involved in amino acid biosynthesis, presumably as precursors for translation of viral products. Similar results have been observed for other phage-host interactions, suggesting that while varied, phage infection strategies centre around reprogramming of host machinery to produce new phage virions (61–65). Chaperones involved in protein folding in response to heat shock (*dnaJ* and *dnaK*) and osmotic stress (*osmY*) were also upregulated. Other studies also report the upregulation of chaperones and other stress-inducible proteins during phage infection (66,67), indicating a general host response to phage stress.

During late infection, the expression of genes involved in chemotaxis and flagella regulation, biosynthesis and structure was decreased (Figure 3D). In addition, the *srfCB* operon encoding a putative virulence factor was also downregulated. Both, up and down regulation of bacterial virulence factors have been reported in different phage-host interactions (68). Phage defence systems encoded in *Serratia* (3 CRISPR-Cas systems, 32 TA systems and one R-M system) remained unaltered by phage infection. Likewise, none of the three prophages present in *Serratia* were upregulated. In summary, phage infection causes major host transcriptional changes during later stages, including changes to metabolism, virulence factors and motility.

### JS26 is sensitive to native type I CRISPR-Cas defence in *Serratia*

**Figure 4.**
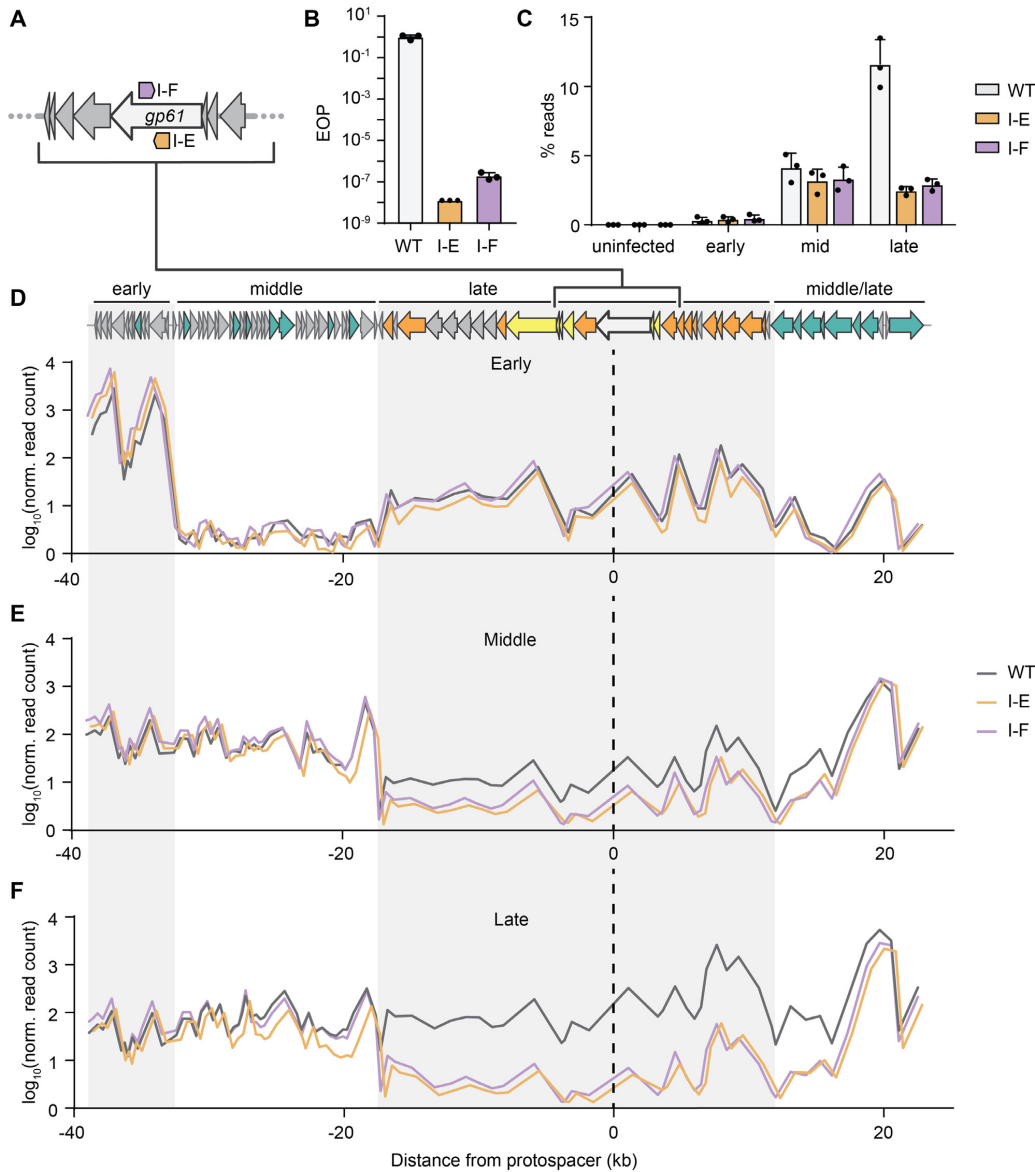
Targeting by type I CRISPR-Cas systems affects transcription of phage genes during mid and late infection. (**A**) Schematic of type I-E and I-F anti-JS26 spacers targeting the tape measure gene (*gp61*). (**B**) EOP assay on strains carrying anti-JS26 spacers in the CRISPR native arrays CRISPR1 (type I-E, PCF524) and CRISPR2 (type I-F, PCF525). (**C**) Percentage of reads mapping to JS26 in strains without (WT, grey) or carrying type I-E (orange) or type I-F (purple) anti-phage spacers during early, middle and late stages of infection. Mean count of reads mapping to each gene across the phage genome during (**D**) early (**E**) mid and (**F**) late infection. In (D)–(F), read count is represented as mean of log_10_(*x* + 1) and distance from protospacer (PS) (black dashed line) was calculated from the midpoint of each gene. The small shift observed for the type I-F targeting strain is due to the distance between the location of the type I-E versus the type I-F protospacer. Grey and white boxes represent clusters of genes expressed during early, middle, middle/late and late stages of infection. In B and C, data are represented as mean of biological triplicates ± SD.

Next, we sought to investigate how CRISPR immunity affects the development of JS26 infection. We generated strains carrying spacers targeting the tape measure gene (*gp61*) in the native type I-E (CRISPR1) and the type I-F (CRISPR2) arrays (Figure 4A). Both CRISPR-Cas systems carrying one or two anti-phage spacers provided robust immunity against JS26 infection (Figure 4B and Supplementary Figure S3A). Strong protection was also observed in liquid culture, even at high moi (Supplementary Figure S3B). Our results show that strains carrying type I anti-JS26 spacers in their chromosomal arrays are resistant to JS26 infection. Strains carrying one anti-phage spacer in each type I system were chosen as representatives for further investigation.

To compare the effects of both type I CRISPR-Cas immune responses on phage JS26, we analysed the transcriptome of immune strains during infection using RNA sequencing. Firstly, the global impact of phage infection was evaluated. Unlike the non-immune *Serratia* WT, interference in both type I JS26 immune strains led to a drop in the abundance of reads mapping to the phage during mid and late infection (Figure 4C and Supplementary Figure S4). To examine the effects of target position on gene expression, we analysed the read distribution across the phage genome in relation to both protospacers (Figure 4D–F). The same transcription profile was observed in the three strains during early infection (Figure 4D). In contrast, genes encoded within ∼20 kb 5′ or 3′ from the targeting site showed a drop in expression from mid to late infection when compared with the WT strain (Figure 4E, F). Thus, our results indicate than rather than a global decrease in total phage transcripts, type I interference will impact the expression of genes encoded in, and neighbouring, the targeted region. Since early phage genes are not encoded in the targeted region and are probably transcribed before interference occurs, their expression remained unaltered even in the presence of immunity. Here we show that targeting by both type I CRISPR-Cas systems in *Serratia* have a similar effect on phage infection, hampering phage gene expression during mid and late infection.

### Phage infection alters the host transcriptional profile even in the presence of CRISPR immunity

**Figure 5.**
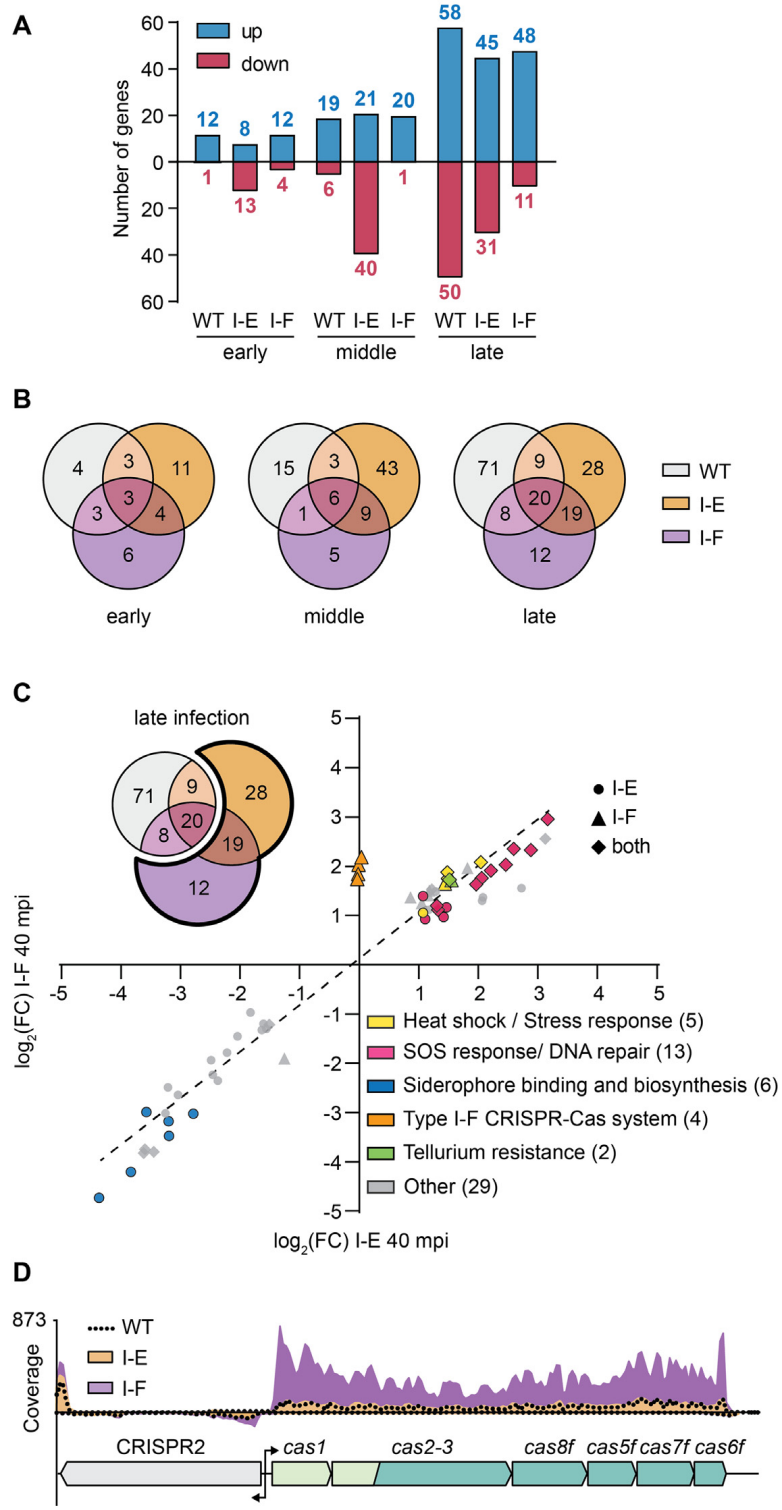
Type I-F immunity induces type I-F *cas* expression. (**A**) Number of genes significantly up and downregulated in *Serratia* and the type I-E and I-F targeting strains. (**B**) Overlapped response of the three strains throughout infection. Total differentially expressed genes in each condition were classified into groups corresponding to the following responses: ‘phage induced’ (pink, genes differentially expressed in the three strains), ‘CRISPR dependent’ (brown, genes differentially expressed in both I-E and I-F targeting strains) and ‘CRISPR type-dependent’ (orange and purple, genes only differentially expressed in either the type I-E or I-F targeting strains). (**C**) Comparison of expression landscape in the type I-E and type I-F targeting strains during late infection. Genes significantly differentially expressed in either the I-E (•, orange), the I-F (▴, purple) or in both strains (✦, brown) were classified by gene ontology. (**D**) Read coverage of the type I-F operon in *Serratia* WT (dotted black line), type I-E (orange) and type I-F (purple) targeting strains. Genes encoding the adaptation complex (light green), interference complex (green) and CRISPR array (grey) are depicted.

To evaluate the bacterial response to phage infection during type I CRISPR-Cas immunity we compared RNA levels at early, mid and late infection (Supplementary Tables S8 and S9). Overall, fewer transcriptional changes were observed in the type I-F immune strain than the type I-E targeting strains (Figure 5A and Supplementary Figure S5A–C). To investigate the responses, transcripts differentially expressed in early, mid and late stages of infection were classified into three distinct groups: the ‘phage induced’ (common to the three strains and irrespective of immunity), ‘CRISPR-dependent’ (for genes significantly differentially expressed in the targeting strains) and ‘CRISPR type-dependent’ response (genes only differentially expressed in either the type I-E or I-F targeting strains) (Figure 5B).

First, genes differentially expressed in the ‘phage induced’ response were examined (Supplementary Tables S8 and S9). Among the hits upregulated during early and middle stage of infection, genes involved in nitrate reductase, Na^+^/H^+^ antiporter and the electron transport chain (Ni/Fe hydrogenase cytochrome β-subunit) were found. Furthermore, during mid and late infection, genes involved in menaquinone biosynthesis, proteolysis and protein folding were also upregulated. In the type I-E, but not type I-F targeting strain, the transcriptional regulator of flagella biosynthesis, *flhC*, was downregulated during late infection. These results suggest that even when the bacterial host is protected by type I CRISPR-Cas targeting, phage infection still alters bacterial physiology.

### Phage immunity by either type I CRISPR-Cas system induces DNA repair and SOS responses

Next, we analysed the CRISPR-dependent response. The majority of genes identified in both targeting strains were involved in DNA repair and recombination (recombination regulator *recX*, DNA polymerase IV, recombinase *recA*, DNA repair protein *recN*, *dinI* family protein, DNA helicase *ruvB*, DNA helicase II), SOS (cell division inhibitor *sulA*, repressor *lexA*), stress and heat-shock response (heat shock protein *hspQ*, heat shock protein *ibpA*) (Figure 5C). Interestingly, genes predicted to be involved in tellurium resistance were also upregulated in both targeting strains. Previous studies have shown that lesions in host DNA produced during interference induce the DNA repair and SOS pathways (69,70). Also, activation of the SOS response has been reported during partial targeting of the DSM3 lysogen in *Pseudomonas aeruginosa* (71). Our results suggest that the infected cell cannot differentiate between self and invading DNA and triggers the SOS response even when phage DNA is being degraded. In summary, interference by both CRISPR-Cas systems lead to the activation of stress responses and the upregulation of SOS and DNA repair pathways.

### Type I-F phage immunity induces type I-F *cas* expression

To determine which genes are modulated by each type I CRISPR-Cas system, we investigated the CRISPR type-dependant response. Overall, the type I-E targeting strain had a larger CRISPR type-dependent response to phage infection than the type I-F immune strain. Out of the 43 genes that form the unique response of the type I-E targeting strain, most of the genes (37 genes) were downregulated (Figure 5A). During mid infection, a general downregulation of transporters and membrane components (19 genes) involved in translocation of sugars, amino acids and iron was observed upon I-E targeting (Supplementary Figure S5E). Our results suggest that while effective, protection by the type I-E CRISPR-Cas system is weaker than type I-F immunity, allowing more phage induced transcriptional changes in the host during mid infection.

In the strain bearing type I-F immunity, fewer host genes were differentially expressed than the I-E strain (Figure 5A and B), and most of the genes differentially expressed follow the same expression trend as the type I-E targeting strain (Figure 5C, Supplementary Figure S5D and E). Interestingly, the type I-F *cas* operon contained the only genes that were specifically differentially expressed in the type I-F targeting strain. Genes involved in adaptation (*cas1* and *cas2-3*) and interference (*cas5f* and *cas8f*) were significantly elevated (Figure 5C and Supplementary Figure S6A). Our results demonstrate that increased levels of type I-F *cas* mRNAs are linked to targeting by pre-existing type I-F CRISPR immunity, as this upregulation did not occur in the *Serratia* strain with no or type I-E immunity (Figure 5D). We propose that in the type I-F system, target recognition triggers the autoregulation of *cas* genes to combat the infection. In turn this would lead to a higher availability of type I-F interference complexes, providing faster phage clearance.

## DISCUSSION

Type I CRISPR-Cas systems are the most abundant in nature and their interference mechanism has been thoroughly described (16,72). Yet, the impact of phage targeting by CRISPR-Cas systems on the infected cell is an aspect of immunity that remains unaddressed. Here, we employed transcriptomics to explore the infection dynamic of a *Serratia* phage and its host in the presence of type I-E or type I-F CRISPR immunity. Our results show that even with CRISPR immunity, phage infection still has a significant effect on the bacterial transcriptome and that phage targeting leads to the upregulation of the SOS and DNA repair pathways. Furthermore, we showed that the type I-F immune response against a targeted phage results in a strong elevation in type I-F *cas* mRNAs, which might enable robust viral clearance.

Rapid transcription of early genes is vital for successful phage proliferation. Previous reports show phages can deplete host DNA and transcripts during early infection (11,62,73,74). In contrast, our results suggest that JS26 has a less virulent infection strategy without an apparent dramatic degradation of host transcripts. JS26 upregulates genes for anaerobic respiration while downregulating genes involved in carbon metabolism, with similar pathways reported to be affected during other phage-host interactions (64,66,75). Host genes involved in glutathione biosynthesis were upregulated during mid and late infection. Other studies show that glutathione is reduced in *Pseudomonas* during infection with two different phages (76) and that some phages harbour genes involved in glutathione metabolism (65,77), suggesting the role of glutathione in oxidative stress protection is important for various phage replication strategies. Furthermore, flagella, which are energetically expensive structures, were also downregulated during late infection. In *Serratia*, the activation of the Rcs pathway that senses membrane stress, has been shown to trigger downregulation of flagella, providing surface protection against JS26 infection (24). We speculate that during infection the host activates an energy-saving program, repressing energy costly and growth-related functions, probably to sustain the overall host response to phage. Alternatively, JS26 might suppresses flagella formation as a superinfection immune strategy, preventing free sister phages, or other flagellatropic *Serratia* phages in the environment from adsorbing to the cell. An analogous effect has been shown for phages PA5oct and DMS3 that causes the downregulation of type IV pili in *Pseudomonas*, necessary for sister phage adsorption (9,78).

CRISPR-Cas immunity has been shown to provide protection against mobile genetic elements through different mechanisms. While some CRISPR-Cas systems lead to survival of the infected cells after phage clearance (79,80), others provide population protection by induction of dormancy or death of infected cells (27,29,81). Our results show that both *Serratia* type I CRISPR-Cas systems can provide strong JS26 immunity even at high phage densities, demonstrating that infected cells survive phage infection. Although protection is achieved, the effects of interference on phage gene expression are only observed during mid-late infection. We predict that phage proteins expressed in early infection are responsible for the prevalence of phage induced transcriptomic changes in the host even in the presence of CRISPR immunity. It is also known that type I Cas complexes take time to find their DNA targets in the cell (82), allowing phage transcription to initiate in early-mid infection. Future studies should seek to explore the effects of targeting regions encoding early expressed genes and its impact on the host transcriptome.

Another factor that plays a role in CRISPR-mediated immunity is the number of Cas complexes available in the cell. A recent study has estimated that a single Cas complex takes 1.5 hours to locate a DNA target *in vivo* and that CRISPR protection increases with Cas complex copy number (82). In agreement, other studies have shown that upregulation of CRISPR-Cas loci leads to enhanced interference (83,84). Reports have shown that viral infection can induce the upregulation of defence systems, including CRISPR-Cas systems in bacteria and archaea (7,85–87). Yet, our results indicate that the type I-F operon in *Serratia* is only upregulated in the presence of type I-F immunity, suggesting an auto-regulation mechanism. Similarly, the expression of the type I-A CRISPR-Cas system in *Sulfolobus islandicus* is repressed by binding of the Cas complex and the Csa3b regulator to the promoter region upstream the interference operon (88). Recently, a long-form transactivating crRNA was shown to serve as a guide that directs Cas9 to repress its own promoter, in the type II-A CRISPR-Cas system of *Streptococcus pyogenes* (89). Whether regulation of type I-F CRISPR-Cas system in *Serratia* shows a similar mechanism, or is mediated by another pathway, is yet to be determined.

Overall, our study provides new insights into the physiology of phage infected cells whilst eliciting native CRISPR-Cas immunity. Although type I CRISPR-Cas provides robust immunity, it is unable to provide complete attenuation of phage-induced cellular stress. When more virulent phages infect, and type I CRISPR-Cas immunity struggles to completely clear infection, the bacterial population can still obtain immunity through abortive infection (81). It is likely that the physiological response upon CRISPR-Cas immunity in native phage-host systems displays a spectrum that is dependent of phage virulence and CRISPR-Cas immune strength.

## DATA AVAILABILITY

The DNA sequencing reads from JS26 and the raw reads from the RNA-sequencing experiment have been deposited in the NCBI SRA with the BioProject number PRJNA746619. Processed data files have been deposited in GEO under accession number GSE186673. The JS26 genome has been deposited in the NCBI GenBank database with the accession number NC_053012.1. The mass spectrometry proteomics data have been deposited to the ProteomeXchange Consortium via the PRIDE (90) partner repository with the dataset identifier PXD029878.

## Supplementary Material

gkab1210_Supplemental_File
