# Supplementary material for: Type I CRISPR-Cas provides robust immunity but incomplete attenuation of phage-induced cellular stress
Source: Nucleic Acids Res. 2021 Dec 20;50(1):160–74. doi: 10.1093/nar/gkab1210 (PMC8754663; doi:10.1093/nar/gkab1210)
Supplement: gkab1210_Supplemental_File [file gkab1210_supplemental_file.pdf]

**Supplementary data**

# Type I CRISPR-Cas provides robust immunity but incomplete attenuation of phage-induced cellular stress

**Lucia M. Malone[1], Hannah G. Hampton[1], Xochitl C. Morgan[1] and Peter C. Fineran[1*]**

[1]Department of Microbiology and Immunology, University of Otago, PO Box 56, Dunedin 9054, New Zealand.

***Author to whom correspondence should be addressed; e-mail: peter.fineran@otago.ac.nz

## Supplementary figures

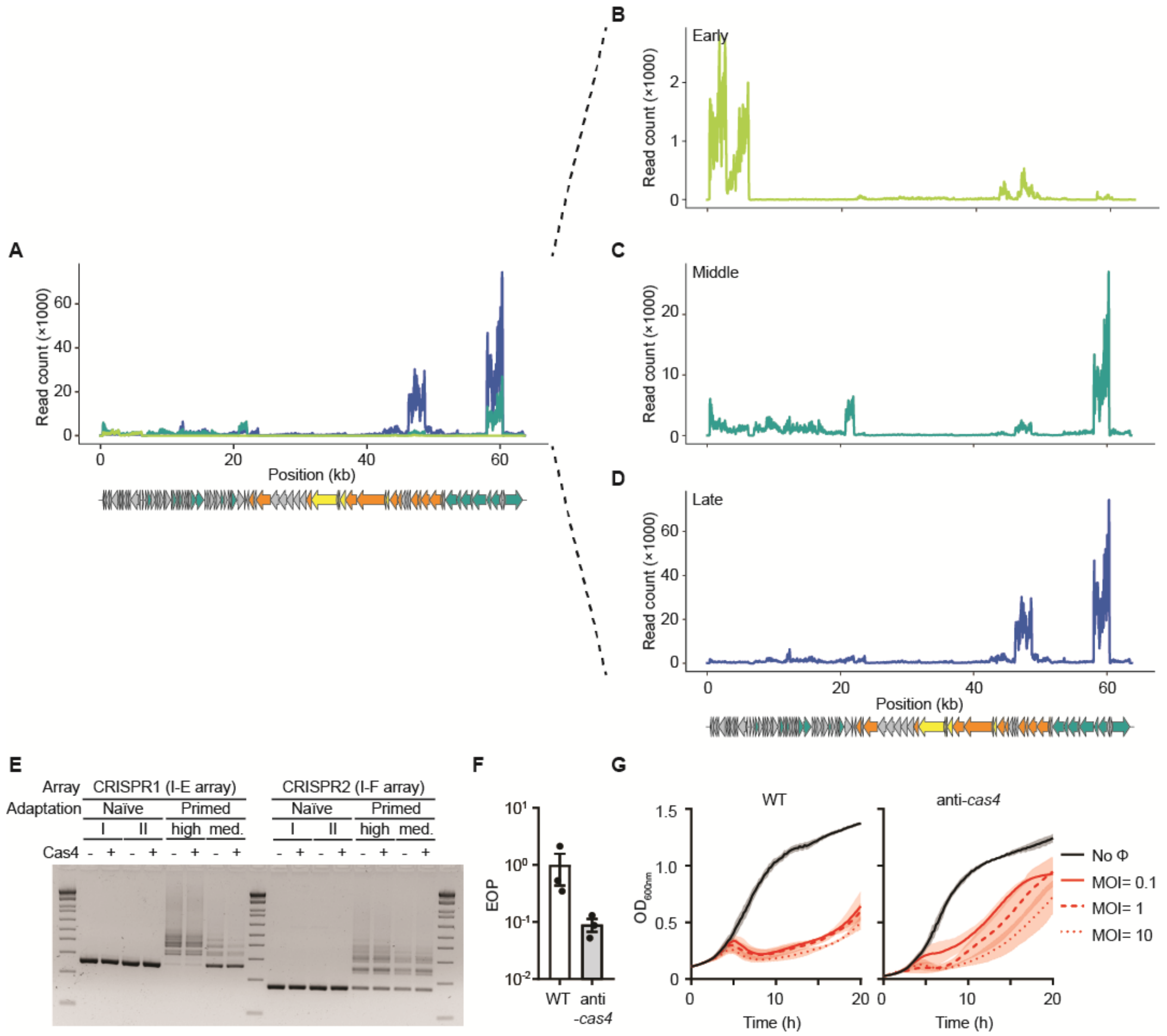


**Figure S1. Phage genes are highly expressed during late infection. A.** RNA read density plot across the JS26 genome at **B.** early (5 mpi), **C.** mid (20 mpi) and **D.** late (40 mpi) infection. For ease of visualisation, reads from either strand are shown in a single plot (note: no reads mapped to non-coding regions). One representative replicate per time point is shown. **E.** Type I-E and I-F array expansion during naïve (I, pPF953 and II, pPF719) and primed CRISPR adaptation (type I-E array expansion: high, pPF1233; medium, pPF1048 and type I-F array expansion: high, pPF1242; medium: pPF1243) during *cas4* overexpression (pPF2291, empty vector: pPF783) after 3 days of passaging. **F.** Plaquing efficiency and **G.** infection in liquid culture of *Serratia* with (anti-*cas4*, pPF2786) or without (WT, pPF1755, empty vector) CRISPR-dCas9 silencing vector. In E, F and G experiments were performed in three biological triplicates.

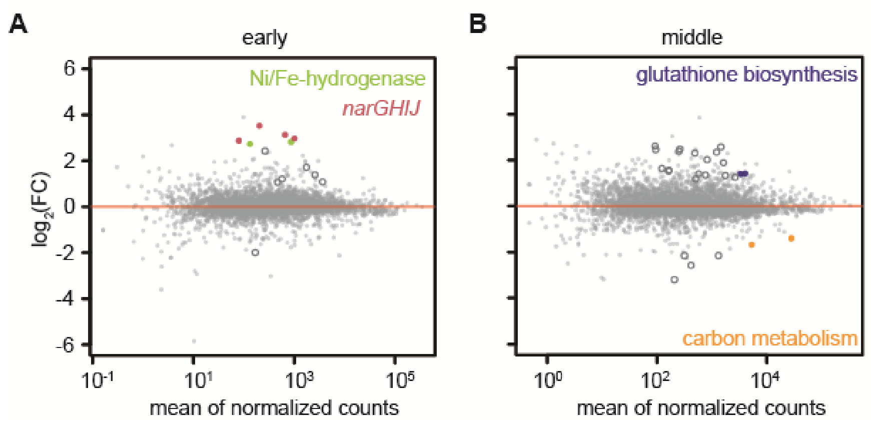


**Figure S2. The host has a small early and mid response to phage infection.** Expression landscape in *Serratia* during **A.** early (5 mpi) and **B.** mid (20 mpi) infection. Genes significantly differentially expressed ($log_2$(FC)>1.5 and padj<0.05) are represented as coloured dots or empty circles.

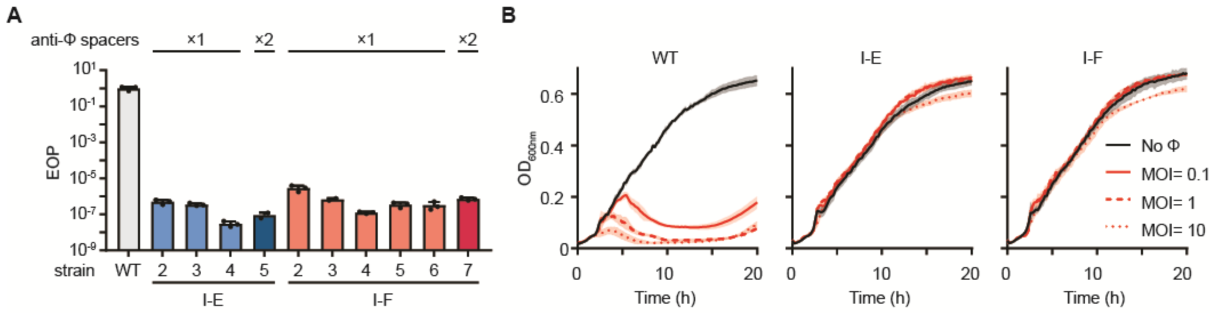


**Figure S3. Type I CRISPR-Cas systems provide robust immunity against JS26.A.** EOP assay on strains carrying anti-JS26 spacers in the CRISPR native arrays CRISPR1 (I-E) and CRISPR2 (I-F). Anti-JS26 I-E strains: 2 (PCF552), 3 (PCF554), 4 (PCF556), 5 (PCF558, ×2 anti-JS26 spacers) and anti-JS26 I-F strains: 2 (PCF562), 3 (PCF568), 4 (PCF574), 5 (PCF577), 6 (PCF579), 7 (PCF580, ×2 anti-JS26 spacers). **B.** Plate reader assay for *Serratia* WT and strains with anti-JS26 spacers in the I-E (PCF524) and I-F (PCF525) array uninfected (moi=0) or infected with JS26 (moi=0.1, 1 and 10). Data are represented as mean of biological triplicates ± SD (n=3).

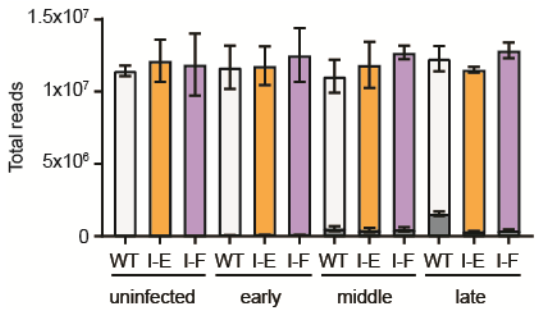


**Figure S4. CRISPR immunity results in a reduced number of reads mapping to JS26 during late infection.** Total number of reads mapping to *Serratia* WT (pale grey) and strains carrying anti-phage spacers in the I-E (orange, PCF524), and I-F (purple, PCF525) CRISPR arrays during early (5 mpi), mid (20 mpi) and late (40 mpi) infection. Total number of reads mapping to JS26 are represented as dark grey bars.

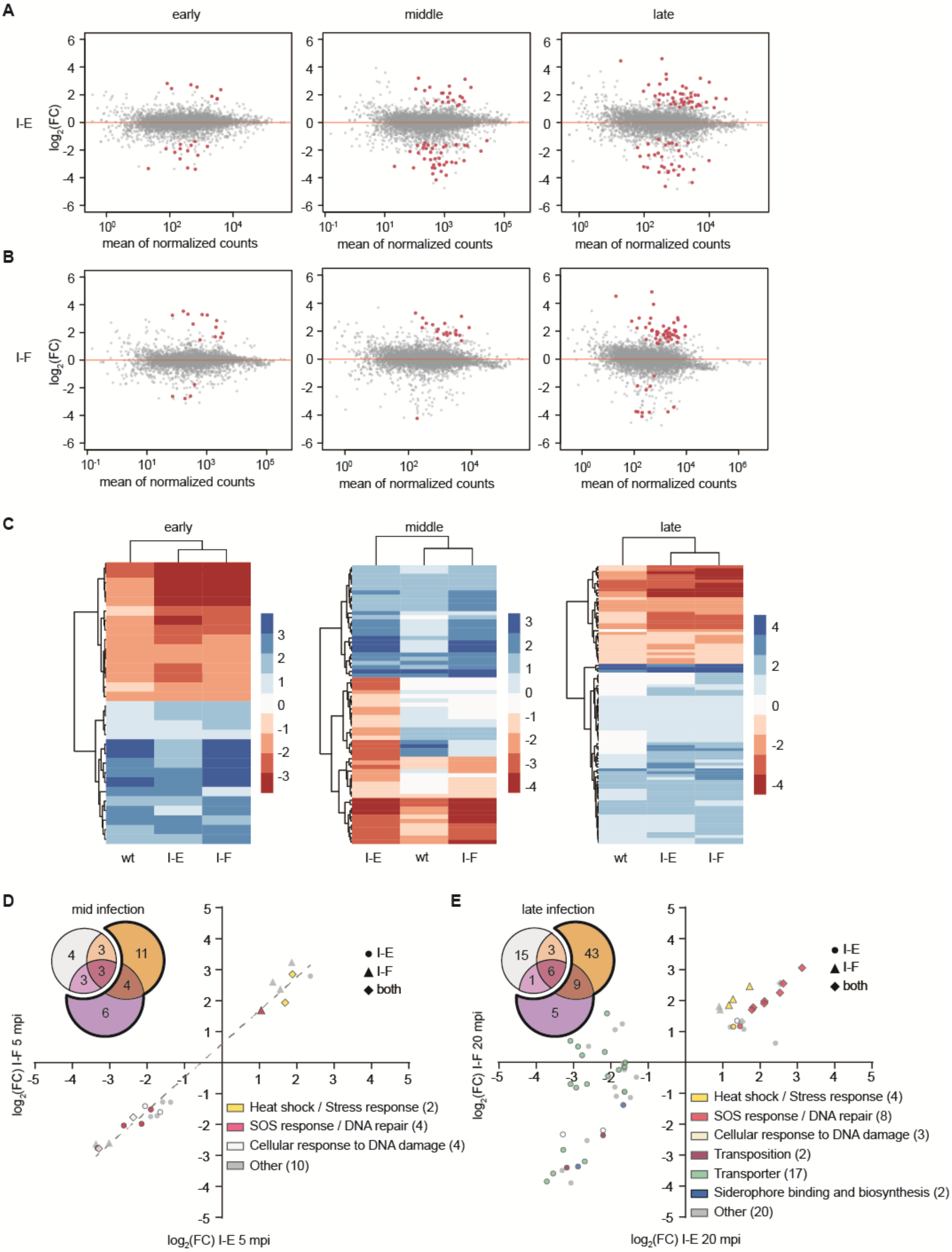


**Figure S5. Type I-E and I-F targeting results in different transcriptomic landscapes.** Expression landscape in **A.** type I-E and **B.** type I-F targeting strains during early, mid and late infection. Genes significantly differentially expressed ($\log_2$(FC)>1.5 and padj<0.05) are represented as red dots. **C.** Heat map of significantly differentially expressed genes in *Serratia* WT and type I-E and I-F targeting strains throughout infection. Comparison of expression landscape in the type I-E and type I-F targeting strains during **D.** early and **E.** mid infection. Number of genes significantly differentially expressed only in the I-E (●, orange in Venn

diagram), the I-F (▲, purple in Venn diagram) or in both strains (◆, brown in Venn diagram) were classified into gene ontology.

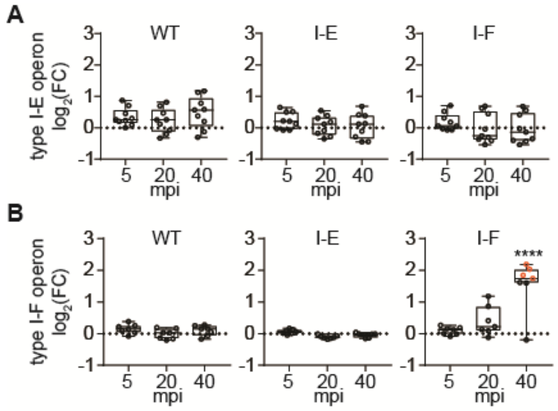


**Figure S6. The type I-F CRISPR-Cas operon is upregulated upon type I-F targeting.** Expression profile of genes encoded in the **A.** type I-E and **B.** type I-F CRISPR-Cas operon in *Serratia* WT and type I-E and type I-F targeting strains throughout infection. Genes significantly upregulated are represented as red dots. Statistical significance was calculated using a two-way ANOVA and a Tukey's test for multiple comparison. **** indicates a padj<0.0001.

## Supplementary tables

## Table S1. Primer list

| Name | Sequence | Description/ Binding site |
|---|---|---|
| **General cloning** | | |
| PF2296 | TTTTACTAGTGATCGCCATCGCAATCTGAC | JS26 Tape measure gene with SpeI site fwd |
| PF2297 | TTTTGGTACCAGTCCGGGCGTACCAATCAG | JS26 Tape measure gene with KpnI site rev |
| PF1403 | AGCTCCTGAAAATCTCGATAAC | Screening primer pPF1257/58 fwd |
| PF1732 | ATAAGATCTTGTTATCCGCTCACAAAGC | Screening primer pPF1257/58 rev |
| PF4692 | GTTTTTTTGGGCTAGCGAATTCAAAGAGGAGAAATTAACTATGTCCCGCGGGGTGAC | *cas4* (*gp81*) + RBS, overlap with pPF783 (EcoRI) for pPF2291 fwd |
| PF4693 | GTCATCCACTGCAGCATGCTTAATCTTCTTCGCCTTCTTCTTC | *cas4* (*gp81*), overlap with pPF783 (SphI) for construction of pPF2291 rev |
| PF2346 | CTGCAAAAATGCAGTAATATCAAGAAGTTTTACATTGATTGGCTCCAATTCTTGGAG | Protospacer targeted by spacer 1 in CRISPR2 for pPF1242/43 fwd |
| PF2348 | TTTTCCATGGTCTATTCCTGCAAAAATGCAGTAATATCAAG | Protospacer targeted by spacer 1 in CRISPR2 for pPF1242 (GAA_PAM) rev |
| PF2349 | TTTTCCATGGTCTATGCCTGCAAAAATGCAGTAATATCAAG | Protospacer targeted by spacer1 in CRISPR2 for pPF1243 (GCA_PAM) rev |
| **CRISPR expansion PCRs** | | |
| PF1989 | TAAGTTAGTGTTCTTTAACAAGCAGGA | CRISPR1 expansion screening fwd |
| PF1887 | GTTAAGTCAGCAGGCGTTTAGTCG | CRISPR1 expansion screening rev |
| PF633 | GTGGATCTGGATGGACTGC | CRISPR1 expansion screening fwd |
| PF2177 | TTACTGAATTCAGGCGTTTCGTCGTTTTGTG | CRISPR1 expansion screening rev |
| PF1990 | CACGAAAATGATAATTGATGCTGAT | CRISPR2 expansion screening fwd |
| PF1888 | CATCTGATGCTGACGACACTG | CRISPR2 expansion screening rev |
| **Transposon mutagenesis / Arbitrary PCR** | | |
| PF106 | GACCACACGTCGACTAGTGCNNNNNNNNNNAGAG | Random primed PCR primer 1 |
| PF107 | GACCACACGTCGACTAGTGCNNNNNNNNNNACGCC | Random primed PCR primer 2 |
| PF108 | GACCACACGTCGACTAGTGCNNNNNNNNNNGATAC | Random primed PCR primer 3 |
| PF109 | GACCACACGTCGACTAGTGC | Random primed PCR adapter primer |
| PF226 | CATAAGGGACTCCTCATTAAG | Nested primer |
| PF1212 | CGGGAATTCTCATGTTTGAC | Nested primer |
| **RNA extraction DNA contamination screening** | | |
| PF796 | ATAGAATTCAGGAGGAATATAATGGGTACTTCTGAGTTACTTAAGC | *flhDC* forward |
| PF797 | GATCCCGGGTCAGACTGCGTGTTTTACTTG | *flhDC* reverse |

## Table S2. Plasmid list

| Name | Features | Description | Construction | Reference |
|---|---|---|---|---|
| **Priming vectors** | | | | |
| pPF1125 | RP4/oriT, pBR322_origin, CmR, mCherry, lacI/T5 | Phage priming vector with I-E PPS (spacer 1 CGT PAM) | | (1) |
| pPF1126 | RP4/oriT, pBR322_origin, CmR, mCherry, lacI/T5 | Phage priming vector with I-F PPS (spacer 2 GT PAM) | | (1) |
| pPF1257 | RP4/oriT, pBR322_origin, CmR, mCherry, lacI/T5 | Phage priming vector with I-E PPS (spacer 1 CGT PAM) with tail tape measure gene insert (*gp61*) | PF2296/PF2297 into pPF1125 (SpeI, KpnI) | This study |
| pPF1258 | RP4/oriT, pBR322_origin, CmR, mCherry, lacI/T5 | Phage priming vector with I-F PPS (spacer 2 GT PAM) with tail tape measure gene insert (*gp61*) | PF2296/PF2297 into pPF1126 (SpeI, KpnI) | This study |
| ***cas4* heterologous expression** | | | | |
| pPF783 | OriT, p15A, GmR, AraC | Expression vector | | Jackson, unpublishe d |
| pPF2291 | OriT, p15A, GmR, AraC | *cas4* (*gp81*) expression vector | PF4692/PF4693 (JS26) into pPF783 (EcoRI, SphI) | This study |
| **Adaptation assay** | | | | |
| pPF719 | RP4/oriT, pBR322_origin, TcR, mCherry, lacI/T5 | Naïve control priming plasmid (no protospacer) | | (2) |
| pPF953 | RP4/oriT, pBR322_origin, TcR, mCherry, lacI/T5 | Naïve control priming plasmid (no protospacer) | | (1) |
| pPF1233 | RP4/oriT, pBR322_origin, TcR, mCherry, lacI/T5 | Type I-E priming plasmid containing protospacer from CRISPR1 spacer 1 (CGT_PAM) | | (3) |
| pPF1048 | RP4/oriT, pBR322_origin, TcR, mCherry, lacI/T5 | Type I-E priming plasmid containing protospacer from CRISPR1 spacer 1 (TCA_PAM) | | (2) |
| pPF1242 | RP4/oriT, pBR322_origin, TcR, mCherry, lacI/T5 | Type I-F priming plasmid containing protospacer from CRISPR2 spacer 1 (GAA_PAM) | PF2346/PF2348 into pPF953 (NcoI/XhoI) | Jackson, unpublishe d |
| pPF1243 | RP4/oriT, pBR322_origin, TcR, mCherry, lacI/T5 | Type I-F priming plasmid containing protospacer from CRISPR2 spacer 1 (GCA_PAM) | PF2346/PF2348 into pPF953 (NcoI/XhoI) | Jackson, unpublishe d |
| **CRISPRi** | | | | |
| pPF1755 | oriT, RK2, KmR, AraC, dCas9 | dCas9 HF expression vector | | Jackson and Yin, unpublishe d |
| pPF2786 | oriT, RK2, KmR, AraC, dCas9 | dCas9 HF expression vector with gRNA targeting *cas4* (*gp81* in JS26) | pPF1755 (BsaI) + PF5506/PF5507 | Malone and Harding, unpublishe d |

## Table S3. Strain list

| Species | Strain | Description | Notes | Construction | Reference |
|---|---|---|---|---|---|
| *E. coli* | DH5α | Cloning strain | | | Gibco/BRL |
| *E. coli* | ST18 | Auxotrophic donor for biparental conjugation | Requires ALA | | (4) |
| *Serratia* sp. ATCC 39006 | LacA | *lac* EMS mutant, denoted WT | | | (5) |
| **Receptor mutants** | | | | | |
| *Serratia* sp. ATCC 39006 | PCF619 | *flgI*::mini-Tn5, flagella mutant | KmR | transposon mutagenesis | This study |
| *Serratia* sp. ATCC 39006 | PCF620 | *flhD*::mini-Tn5, flagella mutant | KmR | transposon mutagenesis | This study |
| *Serratia* sp. ATCC 39006 | PCF621 | *fliR*::mini-Tn5, flagella mutant | KmR | transposon mutagenesis | This study |
| *Serratia* sp. ATCC 39006 | PCF623 | *flhC*::mini-Tn5, flagella mutant | KmR | transposon mutagenesis | This study |
| *Serratia* sp. ATCC 39006 | PCF879 | Δ*flhDC*, flagella master transcriptional regulator knock-out | CmR | | (6) |
| **Strains with spacers in native CRISPR arrays** | | | | | |
| *Serratia* sp. ATCC 39006 | PCF524 | anti-JS26 spacer in type I-E system | | plasmid loss with pPF1257 | This study |
| *Serratia* sp. ATCC 39006 | PCF552 | anti-JS26 spacer in type I-E system | | plasmid loss with pPF1257 | This study |
| *Serratia* sp. ATCC 39006 | PCF554 | anti-JS26 spacer in type I-E system | | plasmid loss with pPF1257 | This study |
| *Serratia* sp. ATCC 39006 | PCF556 | anti-JS26 spacer in type I-E system | | plasmid loss with pPF1257 | This study |
| *Serratia* sp. ATCC 39006 | PCF558 | anti-JS26 spacer in type I-E system | | plasmid loss with pPF1257 | This study |
| *Serratia* sp. ATCC 39006 | PCF525 | anti-JS26 spacer in type I-F system | | plasmid loss with pPF1258 | This study |
| *Serratia* sp. ATCC 39006 | PCF562 | anti-JS26 spacer in type I-F system | | plasmid loss with pPF1258 | This study |
| *Serratia* sp. ATCC 39006 | PCF568 | anti-JS26 spacer in type I-F system | | plasmid loss with pPF1258 | This study |
| *Serratia* sp. ATCC 39006 | PCF574 | anti-JS26 spacer in type I-F system | | plasmid loss with pPF1258 | This study |
| *Serratia* sp. ATCC 39006 | PCF577 | anti-JS26 spacer in type I-F system | | plasmid loss with pPF1258 | This study |
| *Serratia* sp. ATCC 39006 | PCF579 | anti-JS26 spacer in type I-F system | | plasmid loss with pPF1258 | This study |
| *Serratia* sp. ATCC 39006 | PCF580 | anti-JS26 spacer in type I-F system | | plasmid loss with pPF1258 | This study |

**Table S4. Spacers in native CRISPR arrays**

| Strain name | a.k.a. | No. of new spacers | Sequence | Protospacer |
|---|---|---|---|---|
| PCF524 | I-E_1 | 1 | AAACTCGGCGGTACCGAGGGCGCGGCCTTCCA | Phage tape measure gene |
| PCF554 | I-E_3 | 4 | TAGGCCCACCCGCGAGCGGGTGTTCCTTCTTC | oriT |
| | | | GCCACGCCCACCGAGTCAGCGGCACCCTTGAT | Phage tape measure gene |
| | | | CGTGGCGCTTTCTCATAGCTCACGCTGTAGGT | pBR322_origin |
| | | | GGTGAACACTATCCCATATCACCAGCTCACCG | Cm resistance |
| PCF556 | I-E_4 | 3 | GCCGCTTTGTTGGCCTCCGCAAACTGGCGAAT | Phage tape measure gene |
| | | | CGTGGCGCTTTCTCATAGCTCACGCTGTAGGT | pBR322_origin |
| | | | GGTGAACACTATCCCATATCACCAGCTCACCG | Cm resistance |
| PCF558 | I-E_5 | 5 | GCGCGTGCTGCCGAGGACGGCGGGAAGGCTG A | Phage tape measure gene |
| | | | AGTGGAAGCGGCGATGGCGGAGCTGAATTACA | *lacI* |
| | | | ATTGCAGCGATCGAGCAGCAACTGCAGGCGGC | Phage tape measure gene |
| | | | CGTGGCGCTTTCTCATAGCTCACGCTGTAGGT | pBR322_origin |
| | | | GGTGAACACTATCCCATATCACCAGCTCACCG | Cm resistance |
| PCF525 | I-F_1 | 1 | TGATACTCGTCGAGGTTAGCCATCAGGGTGTT | Phage tape measure gene |
| PCF562 | I-F_2 | 1 | ACGATCGAGTCGAAGGCCACGCCCACCGAGTC | Phage tape measure gene |
| PCF568 | I-F_3 | 2 | TTGATGCCCATCTGCAGGTAGTCGTTGAGTTC | Phage tape measure gene |
| | | | ACTTCAAGAACTCTGTAGCACCGCCTACATAC | pBR322_origin |
| PCF574 | I-F_4 | 2 | GAACATATMGGACCATTMGGCAGTCCCGTTCT | Phage tape measure gene |
| | | | AGTTWTGATTTAAACGTGGCCAATATGGACAA | Cm resistance |
| PCF577 | I-F_5 | 1 | GGTAATGGCACCCATGTCGCCCTGCAGCGCTT | Phage tape measure gene |
| PCF579 | I-F_6 | 2 | CAGGACTCCTCCCTGCAGGACGGCGAGTTCAT | mCherry |
| | | | ATTTTATCGCCCCGGGTTGTCAGCCGGTCACG | Phage tape measure gene |
| PCF580 | I-F_7 | 3 | AATGTGCTGTTAAGCAGCAGCGCCTGGTCCCTC | Phage tape measure gene |
| | | | TGGGCCAACTTTTGGCGAAAATGAGACGTTGA | plasmid backbone |
| | | | CTTGATGCCCATCTGCAGGTAGTCGTTGAGTT | Phage tape measure gene |

## Table S5. JS26 genome annotation

| ORF | Protein function | Start (bp) | Stop (bp) | Length (bp) | Proteomic hit[(1)] | Strand |
|---|---|---|---|---|---|---|
| *gp01* | Hypothetical protein CDS | 752 | 895 | 144 | | - |
| *gp02* | Hypothetical protein CDS | 892 | 1218 | 327 | | - |
| *gp03* | Hypothetical protein CDS | 1205 | 1690 | 486 | | - |
| *gp04* | Hypothetical protein CDS | 1693 | 1989 | 297 | | - |
| *gp05* | Hypothetical protein CDS | 1989 | 2972 | 984 | | - |
| *gp06* | Hypothetical protein CDS | 3080 | 3292 | 213 | | - |
| *gp07* | Hypothetical protein CDS | 3279 | 3593 | 315 | | - |
| *gp08* | Hypothetical protein CDS | 3586 | 3756 | 171 | | - |
| *gp09* | DnaA C-terminal domain containing protein CDS | 3758 | 4243 | 486 | | - |
| *gp10* | Hypothetical protein CDS | 4268 | 4543 | 276 | | - |
| *gp11* | Hypothetical protein CDS | 4540 | 4860 | 321 | | - |
| *gp12* | Hypothetical protein CDS | 4895 | 6073 | 1179 | | - |
| *gp13* | Hypothetical protein CDS | 6099 | 6314 | 216 | | - |
| *gp14* | Hypothetical protein CDS | 6676 | 6918 | 243 | | + |
| *gp15* | Hypothetical protein CDS | 7132 | 7422 | 291 | | + |
| *gp16* | NAD-dependent DNA ligase CDS | 7497 | 8003 | 507 | | + |
| *gp17* | Hypothetical protein CDS | 7993 | 8622 | 630 | | + |
| *gp18* | Hypothetical protein CDS | 8612 | 8881 | 270 | | + |
| *gp19* | Phage protein CDS | 8932 | 9294 | 363 | | + |
| *gp20* | Hypothetical protein CDS | 9297 | 9860 | 564 | | + |
| *gp21* | Hypothetical protein CDS | 9853 | 10119 | 267 | | + |
| *gp22* | Phage protein CDS | 10112 | 10900 | 789 | | + |
| *gp23* | Hypothetical protein CDS | 10985 | 11248 | 264 | | + |
| *gp24* | Putative ssDNA binding protein CDS | 11248 | 11775 | 528 | | + |
| *gp25* | Hypothetical protein CDS | 11753 | 12046 | 294 | | + |
| *gp26* | Hypothetical protein CDS | 12043 | 12231 | 189 | | + |
| *gp27* | Nucleoside Triphosphate Pyrophosphohydrolase CDS | 12218 | 12589 | 372 | | + |
| *gp28* | Hypothetical protein CDS | 12636 | 13031 | 396 | | + |
| *gp29* | Hypothetical protein CDS | 13031 | 13384 | 354 | | + |
| *gp30* | Hypothetical protein CDS | 13381 | 13614 | 234 | | + |
| *gp31* | Hypothetical protein CDS | 13611 | 14000 | 390 | | + |
| *gp32* | Putative methylase CDS | 13990 | 14718 | 729 | | + |
| *gp33* | DNA recombination-dependent growth factor C CDS | 14720 | 15844 | 1125 | | + |
| *gp34* | Hypothetical protein CDS | 16013 | 16396 | 384 | | + |
| *gp35* | Hypothetical protein CDS | 16393 | 16671 | 279 | | + |
| *gp36* | Hypothetical protein CDS | 16768 | 17406 | 639 | | + |
| *gp37* | Hypothetical protein CDS | 17406 | 17717 | 312 | | + |
| *gp38* | Hypothetical protein CDS | 17710 | 18429 | 720 | | + |
| *gp39* | Putative Nucleoside 2-deoxyribosyltransferase CDS | 18429 | 18836 | 408 | | + |
| *gp40* | Hypothetical protein CDS | 18907 | 19320 | 414 | | + |
| *gp41* | Hypothetical protein CDS | 19492 | 19800 | 309 | | + |

| ORF | Protein function | Start (bp) | Stop (bp) | Length (bp) | Proteomic hit | Strand |
|---|---|---|---|---|---|---|
| *gp42* | Hypothetical protein CDS | 19800 | 20045 | 246 | | + |
| *gp43* | N-6-adenine-methyltransferase CDS | 20042 | 20749 | 708 | | + |
| *gp44* | Phage protein CDS | 20884 | 21921 | 1038 | | + |
| *gp45* | Hypothetical protein CDS | 21936 | 22124 | 189 | | + |
| *gp46* | Phage protein CDS | 22284 | 22553 | 270 | | - |
| *gp47* | Phage capsid and scaffold CDS | 22550 | 23314 | 765 | x | - |
| *gp48* | Hypothetical protein CDS | 23326 | 23655 | 330 | | - |
| *gp49* | Putative capsid protein CDS | 23663 | 25786 | 2124 | x | - |
| *gp50* | Hypothetical protein CDS | 25796 | 27043 | 1248 | x | - |
| *gp51* | Hypothetical protein CDS | 27054 | 28199 | 1146 | x | - |
| *gp52* | Phage protein CDS | 28212 | 29228 | 1017 | x | - |
| *gp53* | Phage protein CDS | 29241 | 30191 | 951 | x | - |
| *gp54* | Phage protein CDS | 30194 | 31156 | 963 | x | - |
| *gp55* | Tail fiber protein CDS | 31156 | 31890 | 735 | x | - |
| *gp56* | Putative tail assembly protein CDS | 31902 | 35720 | 3819 | x | - |
| *gp57* | Putative tail assembly protein CDS | 35707 | 35928 | 222 | | - |
| *gp58* | Putative tail assembly protein CDS | 35928 | 36170 | 243 | | - |
| *gp59* | Putative tail assembly protein CDS | 36181 | 37002 | 822 | x | - |
| *gp60* | Putative distal tail protein | 37013 | 38698 | 1686 | x | - |
| *gp61* | Putative tail length tape measure protein CDS | 38709 | 42851 | 4143 | x | - |
| *gp62* | Putative tail assembly protein CDS | 42844 | 43089 | 246 | | - |
| *gp63* | Putative tail assembly protein CDS | 43086 | 43547 | 462 | | - |
| *gp64* | Putative tail protein CDS | 43677 | 44816 | 1140 | x | - |
| *gp65* | Putative minor tail protein CDS | 44829 | 45371 | 543 | x | - |
| *gp66* | Phage protein CDS | 45368 | 45985 | 618 | x | - |
| *gp67* | Phage protein CDS | 45987 | 46361 | 375 | x | - |
| *gp68* | hypothetical protein CDS | 46364 | 46735 | 372 | | - |
| *gp69* | Phage major capsid protein CDS | 46799 | 47863 | 1065 | x | - |
| *gp70* | Capsid decoration protein CDS | 47874 | 48275 | 402 | x | - |
| *gp71* | Head-tail preconnector protein GP5 CDS | 48286 | 49575 | 1290 | x | - |
| *gp72* | Phage portal protein CDS | 49581 | 51284 | 1704 | x | - |
| *gp73* | Head-tail joining protein CDS | 51284 | 51538 | 255 | x | - |
| *gp74* | Hypothetical protein CDS | 51549 | 51857 | 309 | | - |
| *gp75* | Terminase large subunit CDS | 51972 | 53630 | 1659 | | - |
| *gp76* | Terminase small subunit CDS | 53614 | 54240 | 627 | | - |
| *gp77* | DNA helicase, phage-associated CDS | 54240 | 55727 | 1488 | | - |
| *gp78* | VRR-NUC domain protein CDS | 55714 | 56001 | 288 | | - |
| *gp79* | DNA polymerase B region CDS | 55998 | 58064 | 2067 | | - |
| *gp80* | Putative ligase CDS | 58157 | 58741 | 585 | | - |
| *gp81* | Putative Cas4 CDS | 58762 | 60057 | 1296 | | - |
| *gp82* | Hypothetical protein CDS | 60057 | 60458 | 402 | | - |
| *gp83* | Hypothetical protein CDS | 60682 | 60942 | 261 | | + |
| *gp84* | DNA primase/helicase, phage-associated CDS | 60952 | 63522 | 2571 | | + |

(1) Details provided in Table S6.

## Table S6. Proteomic hits

| ORF | Description | # Unique Peptides[1] | # PSMs[2] | Coverage (%)[3] | Sum PEP Score[4] | Score Mascot[5] |
|---|---|---|---|---|---|---|
| *gp47* | Phage capsid and scaffold CDS | 3 | 10 | 15.0 | 17.0 | 150.3 |
| *gp49* | Putative capsid protein CDS | 68 | 1241 | 87.9 | 951.0 | 21469.9 |
| *gp50* | Hypothetical protein CDS | 34 | 1182 | 74.0 | 556.2 | 21306.4 |
| *gp51* | Hypothetical protein CDS | 23 | 1214 | 65.9 | 431.4 | 20354.0 |
| *gp52* | Phage protein CDS | 63 | 2108 | 86.1 | 694.9 | 41213.1 |
| *gp53* | Phage protein CDS | 35 | 1677 | 76.9 | 538.7 | 36132.3 |
| *gp54* | Phage protein CDS | 49 | 1654 | 93.4 | 625.6 | 30050.2 |
| *gp55* | Tail fibre protein CDS | 16 | 483 | 81.1 | 329.0 | 7820.9 |
| *gp56* | Putative tail assembly protein CDS | 152 | 2748 | 82.4 | 1967.1 | 43757.5 |
| *gp59* | Putative tail assembly protein CDS | 28 | 743 | 75.5 | 316.2 | 11922.2 |
| *gp60* | Putative distal tail protein | 49 | 1508 | 64.2 | 630.7 | 25676.0 |
| *gp61* | Putative tape measure protein CDS | 223 | 5751 | 84.2 | 3275.0 | 109573.6 |
| *gp64* | Putative tail protein CDS | 169 | 14174 | 84.4 | 2195.9 | 254994.2 |
| *gp65* | Putative minor tail protein CDS | 18 | 682 | 92.9 | 361.0 | 10805.6 |
| *gp66* | Phage protein CDS | 13 | 291 | 68.8 | 248.7 | 5448.1 |
| *gp67* | Phage protein CDS | 12 | 234 | 83.9 | 152.3 | 4642.6 |
| *gp69* | Phage major capsid protein CDS | 276 | 27045 | 97.2 | 4065.4 | 419874.7 |
| *gp70* | Capsid decoration protein CDS | 101 | 3541 | 100.0 | 1375.0 | 59501.7 |
| *gp71* | Head-tail preconnector protein CDS | 27 | 373 | 54.3 | 297.5 | 6800.3 |
| *gp72* | Phage portal protein CDS | 124 | 4507 | 84.0 | 1751.1 | 79681.0 |
| *gp73* | Head-tail joining protein CDS | 10 | 260 | 98.8 | 110.3 | 5326.1 |

[1]Unique peptides identified per protein. [2]The number of acquired peptide spectra per protein (PSMs - peptide spectral matches), resulting in the identified [3]sequence coverage (coverage (%)). [4]Protein score calculated as the negative logarithms of the Posterior Error Probability values of PSMs. [5]Score calculated by search engine Mascot.

**Table S7. Genes differentially expressed in *Serratia* during phage infection**

| ORF (CWC46_) | Function | GO molecular function | log2(FC) | lfcSE | padj |
|---|---|---|---|---|---|
| **Early infection** | | | | | |
| RS02465 | ribose ABC transporter permease | transport activity | 1.39 | 0.20 | 1.67E-02 |
| RS05850 | NhaA, Na+/H+ antiporter | sodium ion transport / pH regulation | 1.21 | 0.13 | 1.86E-03 |
| RS10625 | Ni/Fe-hydrogenase cytochrome b subunit | unknown | 2.73 | 0.51 | 1.47E-02 |
| RS10630 | cytochrome-c3 hydrogenase | redox activity | 2.82 | 0.53 | 1.47E-02 |
| RS12605 | NarG, nitrate reductase subunit alpha | nitrate reductase activity / electron carrier | 2.97 | 0.64 | 4.90E-02 |
| RS12610 | NarH, nitrate reductase subunit beta | nitrate reductase activity / electron carrier | 3.13 | 0.60 | 1.47E-02 |
| RS12615 | NarJ, nitrate reductase molybdenum cofactor assembly chaperone | nitrate reductase activity / electron carrier | 3.52 | 0.60 | 1.51E-03 |
| RS12620 | NarI, respiratory nitrate reductase subunit gamma | nitrate reductase activity / electron carrier | 2.88 | 0.46 | 1.51E-03 |
| RS16055 | LysR family transcriptional regulator | transcription regulation | 1.72 | 0.27 | 1.54E-02 |
| RS16060 | S-(hydroxymethyl)glutathione dehydrogenase/class III alcohol dehydrogenase | redox activity | 1.08 | 0.13 | 4.90E-02 |
| RS18585 | GalS, HTH-type transcriptional regulator | transcription regulation | -1.99 | 0.34 | 1.52E-02 |
| RS19145 | PhoB, phosphate regulon transcriptional regulatory protein | transcription regulation / phosphorelay signal transduction | 1.06 | 0.12 | 3.04E-02 |
| RS21500 | hypothetical protein | unknown | 2.43 | 0.45 | 1.77E-02 |
| **Mid infection** | | | | | |
| RS00220 | LysR family transcriptional regulator | transcription regulation | 2.48 | 0.44 | 7.18E-03 |
| RS00965 | FadB, fatty acid oxidation complex subunit alpha | fatty acid metabolism | 2.57 | 0.40 | 3.43E-04 |
| RS00970 | FadA, acetyl-CoA C-acyltransferase | fatty acid metabolism | 2.30 | 0.39 | 4.03E-03 |
| RS03770 | PutA, trifunctional transcriptional regulator/proline dehydrogenase/L-glutamate gamma-semialdehyde dehydrogenase | transcription regulation / amino acid biosynthesis | 2.34 | 0.28 | 2.94E-07 |
| RS05275 | iron permease FTR1 | iron transport | -2.16 | 0.40 | 1.92E-02 |
| RS05850 | NhaA, Na+/H+ antiporter | sodium ion transport | 2.02 | 0.22 | 8.06E-08 |
| RS05855 | NhaR, transcriptional activator | transcription regulation / sodium ion transport | 1.35 | 0.15 | 2.24E-04 |
| RS06280 | PdhR, pyruvate dehydrogenase complex transcriptional repressor | transcription regulation / pyruvate dehydrogenase | -1.68 | 0.29 | 2.93E-02 |
| RS06285 | AceE, pyruvate dehydrogenase (acetyl-transferring), homodimeric type | pyruvate dehydrogenase | -1.40 | 0.20 | 1.46E-02 |
| RS07090 | DUF2502 domain-containing protein | unknown | 1.56 | 0.27 | 4.87E-02 |

| ORF (CWC46_) | Function | GO molecular function | log2(FC) | lfcSE | padj |
|---|---|---|---|---|---|
| RS07105 | DUF1107 domain-containing protein | unknown | 1.88 | 0.17 | 1.43E-10 |
| RS07620 | FadE, acyl-CoA dehydrogenase | fatty acid metabolism | 1.40 | 0.21 | 1.84E-02 |
| RS10685 | DUF2002 domain-containing protein | unknown | 2.61 | 0.30 | 5.37E-08 |
| RS10950 | hypothetical protein | unknown | 2.46 | 0.36 | 1.94E-04 |
| RS11860 | glycine zipper 2TM domain-containing protein | outer membrane | 2.39 | 0.46 | 2.21E-02 |
| RS12150 | esterase | carbohydrate metabolism | -2.16 | 0.39 | 1.46E-02 |
| RS15300 | CutC, copper homeostasis protein | copper ion homeostasis | 1.17 | 0.15 | 1.71E-02 |
| RS16060 | FrmA, S-(hydroxymethyl)glutathione dehydrogenase/class III alcohol dehydrogenase | glutathione biosynthesis | 1.41 | 0.20 | 1.46E-02 |
| RS16065 | FghA, S-formylglutathione hydrolase | glutathione biosynthesis | 1.40 | 0.22 | 3.98E-02 |
| RS16520 | LamB/YcsF family protein | carbohydrate metabolism | 1.63 | 0.24 | 4.03E-03 |
| RS18615 | ABC transporter substrate-binding protein | transport | -3.20 | 0.57 | 2.12E-03 |
| RS18620 | TonB-dependent siderophore receptor | siderophore transport | -2.58 | 0.51 | 1.92E-02 |
| RS18680 | MenD, 2-succinyl-5-enolpyruvyl-6-hydroxy-3-cyclohexene-1-carboxylic-acid synthase | menaquinone biosynthesis | 1.33 | 0.18 | 1.46E-02 |
| RS18685 | MenF, isochorismate synthase | menaquinone biosynthesis | 1.24 | 0.14 | 6.43E-04 |
| RS21855 | ABC transporter substrate-binding protein | polyamine transport | 1.52 | 0.24 | 2.59E-02 |
| **Late infection** | | | | | |
| RS00005 | AspA, aspartate ammonia-lyase | TCA cycle | -1.25 | 0.18 | 1.09E-02 |
| RS00015 | anaerobic C4-dicarboxylate transporter | C4-dicarboxylate transport | -1.49 | 0.19 | 2.98E-04 |
| RS01325 | XylR, xylose operon transcription regulator | transcription regulation | 1.25 | 0.19 | 3.09E-02 |
| RS01395 | HAMP domain-containing protein | chemotaxis | -1.40 | 0.23 | 1.94E-02 |
| RS01725 | ABC transporter ATP-binding protein | peptide transport | 1.47 | 0.26 | 2.82E-02 |
| RS01730 | L-Ala-D/L-Glu epimerase | amino acid catabolism | 1.63 | 0.24 | 9.06E-04 |
| RS01970 | carbon starvation protein A | cellular response to starvation | -4.49 | 0.27 | 1.56E-45 |
| RS01975 | phosphoenolpyruvate carboxylase | TCA cycle | 1.22 | 0.10 | 2.46E-08 |
| RS02115 | LigB, NAD-dependent DNA ligase | DNA replication | 1.60 | 0.29 | 2.32E-02 |
| RS02180 | nitrogen regulation protein NR(II) | transcription regulation / phosphorelay signal transduction | -0.98 | 0.12 | 4.57E-02 |
| RS03125 | malate permease | organic anion transport | -3.43 | 0.78 | 1.47E-02 |

| ORF (CWC46_) | Function | GO molecular function | log2(FC) | lfcSE | padj |
|---|---|---|---|---|---|
| RS03335 | GntP family transporter | gluconate transport | -1.41 | 0.24 | 2.82E-02 |
| RS03345 | aldolase | unknown | -1.45 | 0.20 | 1.38E-03 |
| RS03355 | NAD(P)-dependent oxidoreductase | redox activity | -1.41 | 0.25 | 3.64E-02 |
| RS03495 | agmatine deiminase | putrescine biosynthesis | 1.95 | 0.28 | 1.10E-04 |
| RS03720 | ilv operon leader peptide | branched-chain amino acid biosynthesis | 1.78 | 0.35 | 2.82E-02 |
| RS03725 | acetolactate synthase 2 catalytic subunit | branched-chain amino acid biosynthesis | 2.26 | 0.25 | 5.40E-09 |
| RS03730 | acetolactate synthase 2 small subunit | branched-chain amino acid biosynthesis | 1.44 | 0.22 | 5.15E-03 |
| RS03770 | PutA, trifunctional transcriptional regulator/proline dehydrogenase/L-glutamate gamma-semialdehyde dehydrogenase | transcription regulation / amino acid biosynthesis | 2.89 | 0.22 | 5.53E-23 |
| RS05535 | YjeF, bifunctional ADP-dependent NAD(P)H-hydrate dehydratase/NAD(P)H-hydrate epimerase | nucleotide metabolism | 1.79 | 0.35 | 2.63E-02 |
| RS05725 | oxidative stress defence protein | oxidative stress | 1.17 | 0.12 | 2.65E-04 |
| RS05795 | bifunctional aspartate kinase/homoserine dehydrogenase I | L-lysine, L-methionine, L-threonine biosynthesis / amino acid biosynthesis | 1.22 | 0.19 | 3.17E-02 |
| RS05840 | DnaK, molecular chaperone | heat shock response/ protein folding | 1.88 | 0.29 | 9.06E-04 |
| RS05845 | DnaJ, molecular chaperone | heat shock response/ protein folding | 1.77 | 0.29 | 2.86E-03 |
| RS05850 | NhaA, Na+/H+ antiporter | sodium ion transport | 1.95 | 0.17 | 1.60E-12 |
| RS05855 | NhaR, transcriptional activator | transcription regulation / sodium ion transport | 1.07 | 0.14 | 2.32E-02 |
| RS06280 | PdhR, pyruvate dehydrogenase complex transcriptional repressor | transcription regulation / pyruvate dehydrogenase | -1.39 | 0.19 | 1.61E-03 |
| RS06285 | AceE, pyruvate dehydrogenase (acetyl-transferring), homodimeric type | pyruvate dehydrogenase | -1.43 | 0.12 | 9.85E-10 |
| RS06290 | pyruvate dehydrogenase complex dihydrolipoyllysine-residue acetyltransferase | pyruvate dehydrogenase | -1.23 | 0.17 | 6.06E-03 |
| RS07090 | DUF2502 domain-containing protein | unknown | 2.39 | 0.18 | 5.78E-21 |
| RS07105 | DUF1107 domain-containing protein | unknown | 1.94 | 0.21 | 3.50E-08 |
| RS07300 | CysJ, NADPH-dependent assimilatory sulfite reductase flavoprotein subunit | cysteine biosynthesis / amino acid biosynthesis | 1.91 | 0.41 | 4.57E-02 |
| RS09040 | inositol-1-monophosphatase | inositol phosphate dephosphorylation | -1.27 | 0.18 | 8.35E-03 |
| RS09050 | IscR, Fe-S cluster assembly transcriptional regulator | regulation of transcription | 1.66 | 0.22 | 1.40E-04 |
| RS09545 | transmembrane protein | membrane | 1.24 | 0.12 | 2.13E-05 |
| RS09860 | ManC, mannose-1-phosphate guanylyltransferase/mannose-6-phosphate isomerase | LPS biosynthesis | 1.17 | 0.17 | 2.48E-02 |
| RS10405 | acetate kinase | organic acid metabolism | -1.11 | 0.13 | 2.16E-03 |
| RS10615 | HydA, hydrogenase (NiFe) small subunit | redox activity | -1.95 | 0.33 | 2.69E-03 |

| ORF (CWC46_) | Function | GO molecular function | log2(FC) | lfcSE | padj |
|---|---|---|---|---|---|
| RS10685 | DUF2002 domain-containing protein | unknown | 4.10 | 0.24 | 1.57E-46 |
| RS10690 | hypothetical protein | unknown | 3.65 | 0.74 | 2.86E-03 |
| RS10950 | hypothetical protein | unknown | 2.10 | 0.29 | 2.94E-05 |
| RS10955 | sugar ABC transporter permease | sugar transport | 2.22 | 0.32 | 8.23E-05 |
| RS11350 | FlhC, flagellar transcriptional regulator | transcriptional regulation of flagellum assembly | -1.45 | 0.19 | 5.57E-04 |
| RS11375 | HAMP domain-containing protein | chemotaxis | -1.11 | 0.15 | 2.25E-02 |
| RS11380 | CheR, protein-glutamate O-methyltransferase | chemotaxis | -0.95 | 0.11 | 3.32E-02 |
| RS11400 | FlhB, flagellar type III secretion system protein | flagellum assembly | -1.09 | 0.12 | 3.36E-03 |
| RS11430 | FlgB, flagellar basal body rod protein | flagellum assembly | -1.19 | 0.18 | 4.15E-02 |
| RS11540 | FliG, flagellar motor switch protein | flagellum assembly | -1.20 | 0.19 | 3.68E-02 |
| RS11545 | FliF, flagellar basal body M-ring protein FliF | flagellum assembly | -1.11 | 0.16 | 4.36E-02 |
| RS11550 | FliE, flagellar hook-basal body complex protein FliE | flagellum assembly | -1.27 | 0.16 | 1.17E-03 |
| RS11580 | hypothetical protein | unknown | -1.12 | 0.13 | 3.82E-03 |
| RS11585 | DegT/DnrJ/EryC1/StrS family aminotransferase | transaminase activity | -1.13 | 0.16 | 3.10E-02 |
| RS11860 | glycine zipper 2TM domain-containing protein | outer membrane | 3.89 | 0.32 | 2.72E-22 |
| RS12135 | PTS beta-glucoside transporter subunit IIABC | transport | -2.46 | 0.38 | 1.16E-04 |
| RS12140 | 6-phospho-beta-glucosidase | carbohydrate metabolism | -2.77 | 0.35 | 1.68E-07 |
| RS12145 | carbohydrate porin | transport | -3.19 | 0.49 | 1.91E-05 |
| RS12150 | esterase | carbohydrate metabolism | -3.34 | 0.36 | 2.25E-11 |
| RS12155 | hypothetical protein | unknown | -3.00 | 0.47 | 5.99E-05 |
| RS12185 | non-ribosomal peptide synthetase | catalytic activity | -1.47 | 0.25 | 1.94E-02 |
| RS12280 | DUF2091 domain-containing protein | unknown | 1.54 | 0.24 | 5.24E-03 |
| RS12340 | osmotically-inducible lipoprotein OsmB | oxidative stress | 1.72 | 0.28 | 3.91E-03 |
| RS12875 | ShlB/FhaC/HecB family hemolysin secretion/activation protein | protein transport | -1.29 | 0.20 | 1.94E-02 |
| RS13350 | NAD(P)(+) transhydrogenase (Re/Si-specific) subunit alpha | redox activity | 2.02 | 0.31 | 5.57E-04 |
| RS13355 | NAD(P)(+) transhydrogenase (Re/Si-specific) subunit beta | redox activity | 2.35 | 0.39 | 6.84E-04 |
| RS13465 | SfrC, virulence effector protein | virulence factor | -1.13 | 0.15 | 1.50E-02 |

| ORF (CWC46_) | Function | GO molecular function | log2(FC) | lfcSE | padj |
|---|---|---|---|---|---|
| RS13470 | SrfB, virulence protein | virulence factor | -1.15 | 0.17 | 3.55E-02 |
| RS13475 | hypothetical protein | virulence factor | -1.13 | 0.14 | 8.20E-03 |
| RS13630 | MFS transporter | transport | 2.36 | 0.43 | 2.86E-03 |
| RS13785 | DtpA, dipeptide/tripeptide permease | peptide transport | -1.81 | 0.35 | 2.63E-02 |
| RS14540 | 23S rRNA (guanine(745)-N(1))-methyltransferase | rRNA methylation / rRNA processing | -1.15 | 0.16 | 1.77E-02 |
| RS14885 | FkbM family methyltransferase | unknown | -1.10 | 0.15 | 3.52E-02 |
| RS15300 | CutC, copper homeostasis protein | copper ion homeostasis | 1.27 | 0.16 | 2.29E-03 |
| RS15650 | FAA hydrolase family protein | catalytic activity | -1.38 | 0.18 | 1.38E-03 |
| RS15655 | hypothetical protein | organic anion transport | -2.19 | 0.28 | 3.63E-06 |
| RS15675 | hypothetical protein | catalytic activity | 1.56 | 0.30 | 4.31E-02 |
| RS15785 | DmsD, Tat proofreading chaperone | protein folding | 1.53 | 0.20 | 3.75E-04 |
| RS15945 | DUF2593 domain-containing protein | unknown | 1.88 | 0.37 | 2.32E-02 |
| RS16005 | serine hydrolase | proteolysis | 1.28 | 0.16 | 9.18E-04 |
| RS16160 | DUF2807 domain-containing protein | unknown | 1.50 | 0.26 | 2.32E-02 |
| RS16165 | cytochrome P450 | redox activity | 1.63 | 0.25 | 2.29E-03 |
| RS16175 | hypothetical protein | unknown | 1.40 | 0.24 | 3.32E-02 |
| RS16270 | hypothetical protein | unknown | 1.46 | 0.16 | 1.49E-05 |
| RS16275 | ABC transporter ATP-binding protein | transport | 1.39 | 0.24 | 3.90E-02 |
| RS16280 | hypothetical protein | unknown | 1.48 | 0.26 | 2.82E-02 |
| RS16385 | hypothetical protein | unknown | -1.99 | 0.35 | 3.39E-03 |
| RS16390 | hypothetical protein | unknown | -2.19 | 0.48 | 3.78E-02 |
| RS16540 | hypothetical protein | unknown | 1.31 | 0.14 | 1.91E-05 |
| RS17240 | anaerobic C4-dicarboxylate transporter | C4-dicarboxylate transport | -1.41 | 0.22 | 1.09E-02 |
| RS17330 | alanyl-tRNA editing protein | tRNA aminoacylation | -1.75 | 0.27 | 1.09E-03 |
| RS18075 | cytochrome bd-I ubiquinol oxidase subunit I | aerobic electron transport chain | -1.59 | 0.26 | 7.35E-03 |
| RS18615 | ABC transporter substrate-binding protein | transport | -2.49 | 0.51 | 1.09E-02 |
| RS18620 | TonB-dependent siderophore receptor | siderophore transport | -2.18 | 0.42 | 8.42E-03 |

| ORF (CWC46_) | Function | GO molecular function | **log2(FC)** | lfcSE | padj |
|---|---|---|---|---|---|
| RS18660 | MenE, o-succinylbenzoate--CoA ligase | menaquinone biosynthesis | 1.71 | 0.24 | 5.24E-04 |
| RS18665 | MenC, o-succinylbenzoate synthase | menaquinone biosynthesis | 1.63 | 0.27 | 6.06E-03 |
| RS18670 | MenB, 1, 4-dihydroxy-2-naphthoyl-CoA synthase | menaquinone biosynthesis | 1.73 | 0.27 | 1.70E-03 |
| RS18675 | MenH, 2-succinyl-6-hydroxy-2%2C4-cyclohexadiene-1-carboxylate synthase | menaquinone biosynthesis | 1.88 | 0.26 | 1.25E-04 |
| RS18680 | MenD, 2-succinyl-5-enolpyruvyl-6-hydroxy-3-cyclohexene-1-carboxylic-acid synthase | menaquinone biosynthesis | 1.66 | 0.15 | 2.47E-10 |
| RS18685 | MenF, isochorismate synthase | menaquinone biosynthesis | 1.61 | 0.10 | 2.72E-22 |
| RS18725 | NrdA, ribonucleoside-diphosphate reductase subunit alpha | DNA replication | 1.26 | 0.17 | 3.84E-03 |
| RS20365 | IS1 family transposase | transposition | -2.43 | 0.48 | 8.42E-03 |
| RS20700 | PigA, butyryl-CoA dehydrogenase | antibiotic biosynthesis | 1.82 | 0.37 | 3.97E-02 |
| RS21305 | IolD, 3D-(3%2C5/4)-trihydroxycyclohexane-1%2C2-dione acylhydrolase (decyclizing) | inositol catabolism | 1.31 | 0.22 | 4.15E-02 |
| RS21820 | acetolactate synthase AlsS | valine biosynthesis / amino acid biosynthesis | 1.17 | 0.18 | 4.77E-02 |
| RS21855 | hypothetical protein | unknown | 1.64 | 0.23 | 5.00E-04 |
| RS22235 | hypothetical protein | unknown | 1.79 | 0.20 | 6.19E-07 |
| RS22565 | OsmY, molecular chaperone | protein folding | 1.57 | 0.14 | 1.96E-10 |
| RS22725 | MFS transporter | transport | -1.43 | 0.21 | 2.96E-03 |
| RS22750 | LysR family transcriptional regulator | transcriptional regulation | -1.36 | 0.13 | 2.85E-07 |

## Table S8. Genes differentially expressed in *Serratia* I-E anti-JS26 strain

| DE in strains | locus_tag | product | GO molecular function | $log_2$(FC) | lfcSE | padj |
|---|---|---|---|---|---|---|
| **Early infection** | | | | | | |
| wt, I-E, I-F | RS02465 | ribose ABC transporter permease | sugar transport | 1.71 | 0.26 | 9.54E-03 |
| wt, I-E, I-F | RS10625 | Ni/Fe-hydrogenase cytochrome b subunit | anaerobic respiration | 2.82 | 0.54 | 1.32E-02 |
| wt, I-E, I-F | RS12610 | NarH, nitrate reductase subunit beta | nitrate reductase activity | 2.72 | 0.44 | 8.89E-04 |
| wt, I-E | RS12605 | NarG, nitrate reductase subunit alpha | nitrate reductase activity | 2.56 | 0.40 | 8.89E-04 |
| wt, I-E | RS18585 | GalS, HTH-type transcriptional regulator | transcriptional regulation | -3.38 | 0.44 | 4.99E-07 |
| wt, I-E | RS21500 | hypothetical protein | unknown | 2.45 | 0.41 | 3.83E-03 |
| I-E, I-F | RS00965 | FadB, fatty acid oxidation complex subunit alpha | fatty acid metabolism | -2.37 | 0.41 | 8.27E-03 |
| I-E, I-F | RS02455 | ribokinase | D-ribose metabolism | 1.69 | 0.28 | 2.34E-02 |
| I-E, I-F | RS02475 | RbsD, D-ribose pyranase | monosaccharide metabolism | 1.89 | 0.33 | 2.66E-02 |
| I-E, I-F | RS10190 | FadL, long-chain fatty acid transporter | fatty acid transport | -3.29 | 0.41 | 1.11E-07 |
| I-E | RS00220 | LysR family transcriptional regulator | transcriptional regulation | -2.64 | 0.50 | 1.37E-02 |
| I-E | RS00970 | FadA, acetyl-CoA C-acyltransferase | fatty acid metabolism | -2.06 | 0.38 | 2.66E-02 |
| I-E | RS01640 | PtsN, PTS IIA-like nitrogen-regulatory protein | regulation of ion transmembrane transport | -1.74 | 0.31 | 4.37E-02 |
| I-E | RS02470 | RbsA, ribose ABC transporter ATP-binding protein | sugar transport | 2.37 | 0.33 | 1.15E-04 |
| I-E | RS05265 | FCD domain-containing protein | transcriptional regulation | -3.34 | 0.65 | 9.99E-03 |
| I-E | RS07620 | FadE, acyl-CoA dehydrogenase | fatty acid metabolism | -1.65 | 0.28 | 3.17E-02 |
| I-E | RS10010 | GspB, type II secretion system assembly factor | protein secretion | -1.91 | 0.32 | 1.32E-02 |
| I-E | RS13615 | LysR family transcriptional regulator | transcriptional regulation | -1.90 | 0.36 | 4.37E-02 |
| I-E | RS17740 | amino acid adenylation domain protein | nucleotidyltransferase activity | -1.59 | 0.24 | 1.32E-02 |
| I-E | RS19320 | methyl-accepting chemotaxis protein | chemotaxis | -1.37 | 0.20 | 1.67E-02 |
| I-E | RS22460 | sigma-54-dependent Fis family transcriptional regulator | transcriptional regulation | -2.16 | 0.43 | 4.37E-02 |
| **Mid-infection** | | | | | | |
| wt, I-E, I-F | RS05850 | NhaA, Na+/H+ antiporter | sodium transport | 2.13 | 0.30 | 8.18E-05 |
| wt, I-E, I-F | RS05855 | NhaR, transcriptional activator | sodium transport | 1.47 | 0.20 | 1.62E-03 |
| wt, I-E, I-F | RS07090 | DUF2502 domain-containing protein | unknown | 2.30 | 0.31 | 1.49E-05 |

| DE in strains | locus_tag | product | GO molecular function | $\log_2$(FC) | lfcSE | padj |
|---|---|---|---|---|---|---|
| wt, I-E, I-F | RS10685 | DUF2002 domain-containing protein | unknown | 3.20 | 0.44 | 1.31E-06 |
| wt, I-E, I-F | RS18615 | ABC transporter substrate-binding protein | transporter | -4.16 | 0.69 | 7.14E-05 |
| wt, I-E, I-F | RS18685 | MenF, isochorismate synthase | menaquinone biosynthesis | 1.88 | 0.20 | 6.62E-08 |
| wt, I-E | RS05275 | iron permease FTR1 | iron transport | -3.02 | 0.49 | 1.88E-04 |
| wt, I-E | RS07105 | DUF1107 domain-containing protein | unknown | 1.59 | 0.21 | 3.61E-04 |
| wt, I-E | RS18620 | TonB-dependent siderophore receptor | siderophore transport | -3.82 | 0.64 | 9.40E-05 |
| I-E, I-F | RS07645 | DNA polymerase IV | DNA repair / SOS response | 2.12 | 0.30 | 9.40E-05 |
| I-E, I-F | RS08295 | RecA, recombinase | DNA repair / SOS response | 2.53 | 0.39 | 1.79E-04 |
| I-E, I-F | RS08300 | RecX, recombination regulator | DNA repair / SOS response | 2.11 | 0.31 | 1.91E-04 |
| I-E, I-F | RS08850 | RseA, anti-sigma-E factor | anti-sigma binding | 1.51 | 0.17 | 2.08E-05 |
| I-E, I-F | RS09495 | SulA, cell division inhibitor | cellular response to DNA damage stimulus / SOS response | 2.62 | 0.25 | 1.12E-12 |
| I-E, I-F | RS14825 | hypothetical protein | unknown | 2.54 | 0.36 | 1.49E-05 |
| I-E, I-F | RS21055 | RecN, DNA repair protein | DNA repair/ SOS response | 3.12 | 0.30 | 2.78E-13 |
| I-E, I-F | RS21370 | DinI family protein | DNA repair/ SOS response | 1.82 | 0.33 | 1.33E-02 |
| I-E, I-F | RS22350 | LexA, repressor | DNA repair / SOS response | 1.78 | 0.19 | 1.46E-07 |
| I-E | RS01460 | tripartite tricarboxylate transporter substrate binding protein | transport | -2.16 | 0.37 | 2.92E-03 |
| I-E | RS01920 | polysaccharide deacetylase | carbohydrate metabolism | -1.67 | 0.25 | 2.43E-03 |
| I-E | RS01925 | MFS transporter | transport | -1.78 | 0.28 | 2.90E-03 |
| I-E | RS01930 | NAD(P)-dependent oxidoreductase | redox activity | -1.64 | 0.29 | 2.52E-02 |
| I-E | RS03205 | dicarboxylate/amino acid:cation symporter | transporter | -2.08 | 0.42 | 3.29E-02 |
| I-E | RS05985 | DNA polymerase II | DNA repair / SOS response | 1.46 | 0.21 | 3.89E-03 |
| I-E | RS06370 | sugar ABC transporter substrate-binding protein | sugar transport | -2.93 | 0.51 | 7.16E-04 |
| I-E | RS06375 | sugar ABC transporter permease | sugar transport | -3.08 | 0.59 | 3.23E-03 |
| I-E | RS06380 | carbohydrate ABC transporter permease | sugar transport | -2.64 | 0.58 | 2.85E-02 |
| I-E | RS06810 | aldolase | catalytic activity | -3.30 | 0.74 | 1.90E-02 |
| I-E | RS06815 | ATP-grasp domain-containing protein | TCA cycle | -3.07 | 0.59 | 2.92E-03 |
| I-E | RS06820 | LucA, siderophore biosynthesis protein | siderophore biosynthesis | -2.88 | 0.54 | 2.90E-03 |

| DE in strains | locus_tag | product | GO molecular function | $log_2$(FC) | lfcSE | padj |
|---|---|---|---|---|---|---|
| I-E | RS06825 | MFS transporter | transport | -2.70 | 0.62 | 4.99E-02 |
| I-E | RS06850 | IS200/IS605 family transposase | DNA recombination/ transposition | -2.21 | 0.48 | 4.64E-02 |
| I-E | RS08845 | RpoE, RNA polymerase sigma factor | transcriptional regulation / envelope stress response | 1.28 | 0.17 | 2.90E-03 |
| I-E | RS08855 | RseB, sigma-E factor regulatory protein | anti-sigma binding / regulation of polysaccharide biosynthetic process / envelope stress response | 1.20 | 0.16 | 9.81E-03 |
| I-E | RS10855 | ABC transporter ATP-binding protein | transport | -3.54 | 0.61 | 2.21E-04 |
| I-E | RS10860 | ABC transporter permease | transport | -3.28 | 0.56 | 3.23E-04 |
| I-E | RS10865 | ABC transporter permease | transport | -3.72 | 0.56 | 1.04E-05 |
| I-E | RS10870 | EntS, enterobactin transporter | cellular response to DNA damage stimulus / response to antibiotic | -3.29 | 0.59 | 7.39E-04 |
| I-E | RS12165 | SbnA, 2,3-diaminopropionate biosynthesis protein | siderophore biosynthesis | -1.67 | 0.30 | 2.80E-02 |
| I-E | RS13735 | arabinose ABC transporter substrate-binding protein | transport | -1.88 | 0.33 | 7.61E-03 |
| I-E | RS13920 | OppF, oligopeptide ABC transporter ATP-binding protein | protein transport | -1.63 | 0.17 | 1.60E-07 |
| I-E | RS13925 | OppD, oligopeptide ABC transporter ATP-binding protein OppD | protein transport | -1.61 | 0.21 | 2.54E-04 |
| I-E | RS14775 | ferrous iron transport protein A | iron transport / cellular response to DNA damage stimulus | -2.21 | 0.41 | 8.36E-03 |
| I-E | RS16865 | alcohol dehydrogenase | redox activity | -1.66 | 0.32 | 4.99E-02 |
| I-E | RS17020 | hypothetical protein | unknown | -2.64 | 0.53 | 9.81E-03 |
| I-E | RS17315 | cysteine synthase family protein | amino acid biosynthesis | -1.48 | 0.22 | 3.89E-03 |
| I-E | RS17750 | chlorinating enzyme | unknown | -1.89 | 0.25 | 4.76E-05 |
| I-E | RS17755 | hypothetical protein | unknown | -1.64 | 0.29 | 2.29E-02 |
| I-E | RS17760 | EamA/RhaT family transporter | transport | -1.63 | 0.27 | 9.81E-03 |
| I-E | RS17765 | thioesterase | hydrolase activity | -1.85 | 0.27 | 3.61E-04 |
| I-E | RS18320 | glutamate/aspartate ABC transporter substrate-binding protein | amino acid transport | -1.69 | 0.30 | 1.77E-02 |
| I-E | RS18585 | GalS, HTH-type transcriptional regulator | transcriptional regulation | -2.60 | 0.51 | 8.36E-03 |
| I-E | RS18590 | MglB, galactose/glucose ABC transporter substrate-binding protein | sugar transport | -2.77 | 0.39 | 1.42E-05 |
| I-E | RS18595 | MglA, galactose/methyl galactoside ABC transporter ATP-binding protein | sugar transport | -2.88 | 0.58 | 7.85E-03 |
| I-E | RS18600 | MglC, galactose/methyl galactoside ABC transporter permease | sugar transport | -3.08 | 0.62 | 5.42E-03 |
| I-E | RS19145 | PhoB, phosphate regulon transcriptional regulatory protein | transcription regulation | 1.57 | 0.25 | 9.81E-03 |
| I-E | RS20365 | IS1 family transposase | transposition | -3.18 | 0.59 | 1.60E-03 |

| DE in strains | locus_tag | product | GO molecular function | $log_2(FC)$ | lfcSE | padj |
|---|---|---|---|---|---|---|
| I-E | RS21295 | sugar ABC transporter substrate-binding protein | sugar transport | -2.10 | 0.40 | 1.24E-02 |
| I-E | RS21500 | hypothetical protein | unknown | 2.41 | 0.38 | 3.29E-04 |
| I-E | RS22190 | malate dehydrogenase | TCA cycle / anaerobic respiration | -1.32 | 0.20 | 1.82E-02 |
| I-E | RS22235 | RcnB, Nickel/cobalt homeostasis protein | cellular response to DNA damage | 1.39 | 0.23 | 3.42E-02 |
| **Late infection** | | | | | | |
| wt, I-E, I-F | RS05850 | NhaA, Na+/H+ antiporter | sodium transport | 1.75 | 0.26 | 1.24E-03 |
| wt, I-E, I-F | RS07090 | DUF2502 domain-containing protein | unknown | 2.78 | 0.27 | 7.97E-14 |
| wt, I-E, I-F | RS07105 | DUF1107 domain-containing protein | unknown | 2.11 | 0.18 | 3.44E-14 |
| wt, I-E, I-F | RS09545 | YccA, transmembrane protein | negative regulation of apoptosis | 1.47 | 0.26 | 4.01E-02 |
| wt, I-E, I-F | RS09860 | mannose-1-phosphate guanylyltransferase/mannose-6-phosphate isomerase | polysaccharide biosynthetic process | 1.27 | 0.17 | 4.38E-03 |
| wt, I-E, I-F | RS10685 | DUF2002 domain-containing protein | unknown | 4.61 | 0.38 | 3.45E-23 |
| wt, I-E, I-F | RS10690 | hypothetical protein | unknown | 4.45 | 0.76 | 1.04E-04 |
| wt, I-E, I-F | RS11860 | glycine zipper 2TM domain-containing protein | unknown | 3.49 | 0.65 | 1.24E-03 |
| wt, I-E, I-F | RS12140 | 6-phospho-beta-glucosidase | carbohydrate metabolism | -3.40 | 0.60 | 6.41E-04 |
| wt, I-E, I-F | RS12150 | esterase | carbohydrate metabolism | -3.39 | 0.59 | 4.70E-04 |
| wt, I-E, I-F | RS12155 | hypothetical protein | unknown | -3.26 | 0.65 | 4.39E-03 |
| wt, I-E, I-F | RS12340 | OsmB, osmotically-inducible lipoprotein | osmotic stress | 2.25 | 0.39 | 2.20E-03 |
| wt, I-E, I-F | RS15300 | CutC, copper homeostasis protein | copper homeostasis | 1.32 | 0.18 | 4.75E-03 |
| wt, I-E, I-F | RS15785 | DmsD, Tat proofreading chaperone | protein folding | 1.92 | 0.22 | 6.77E-07 |
| wt, I-E, I-F | RS16005 | serine hydrolase | proteolysis | 1.65 | 0.31 | 3.29E-02 |
| wt, I-E, I-F | RS16385 | hypothetical protein | unknown | -2.34 | 0.39 | 1.17E-03 |
| wt, I-E, I-F | RS18680 | MenD, 2-succinyl-5-enolpyruvyl-6-hydroxy-3-cyclohexene-1-carboxylic-acid synthase | menaquinone biosynthesis | 1.75 | 0.32 | 1.89E-02 |
| wt, I-E, I-F | RS18685 | MenF, isochorismate synthase | menaquinone biosynthesis | 1.98 | 0.13 | 1.17E-22 |
| wt, I-E, I-F | RS22235 | hypothetical protein | unknown | 1.99 | 0.26 | 1.47E-05 |
| wt, I-E, I-F | RS22565 | OsmY, molecular chaperone | protein folding / Response to osmotic stress | 1.85 | 0.29 | 1.40E-03 |
| wt, I-E | RS05725 | oxidative stress defence protein | Oxidative stress response | 1.50 | 0.24 | 1.27E-02 |
| wt, I-E | RS05840 | DnaK, molecular chaperone | protein folding | 1.70 | 0.29 | 9.73E-03 |

| DE in strains | locus_tag | product | GO molecular function | $log_2$(FC) | lfcSE | padj |
|---|---|---|---|---|---|---|
| wt, I-E | RS11350 | FlhC, flagellar transcriptional regulator | flagella | -1.47 | 0.24 | 1.68E-02 |
| wt, I-E | RS12145 | carbohydrate porin | carbohydrate transport | -4.62 | 1.19 | 4.21E-02 |
| wt, I-E | RS12280 | DUF2091 domain-containing protein | unknown | 1.61 | 0.30 | 3.35E-02 |
| wt, I-E | RS12875 | ShlB/FhaC/HecB family hemolysin secretion/activation protein | protein transport | -1.23 | 0.19 | 4.01E-02 |
| wt, I-E | RS16390 | hypothetical protein | unknown | -2.45 | 0.40 | 6.41E-04 |
| wt, I-E | RS16540 | hypothetical protein | unknown | 1.74 | 0.29 | 7.71E-03 |
| wt, I-E | RS17330 | alanyl-tRNA editing protein | tRNA modification | -1.49 | 0.26 | 3.29E-02 |
| I-E, I-F | RS01685 | oligopeptidase A | proteolysis | 1.22 | 0.19 | 4.69E-02 |
| I-E, I-F | RS03060 | IbpA, heat shock protein | Heat shock response | 1.49 | 0.26 | 3.61E-02 |
| I-E, I-F | RS04105 | DNA helicase II | DNA repair | 1.31 | 0.21 | 3.29E-02 |
| I-E, I-F | RS07645 | DinB, DNA polymerase IV | DNA repair / SOS response | 2.21 | 0.24 | 1.05E-08 |
| I-E, I-F | RS08295 | RecA, recombinase | DNA repair / SOS response | 2.91 | 0.34 | 3.84E-09 |
| I-E, I-F | RS08300 | RecX, recombination regulator | DNA repair / SOS response | 2.49 | 0.27 | 7.58E-10 |
| I-E, I-F | RS09495 | SulA, cell division inhibitor | SOS response | 2.62 | 0.20 | 8.71E-22 |
| I-E, I-F | RS09530 | HspQ, heat shock protein | Heat shock response | 2.04 | 0.27 | 3.27E-05 |
| I-E, I-F | RS10830 | isochorismate synthase | siderophore biosynthesis | -3.62 | 0.75 | 5.76E-03 |
| I-E, I-F | RS10845 | MbtH family protein | siderophore biosynthesis | -3.60 | 0.71 | 2.91E-03 |
| I-E, I-F | RS10855 | ABC transporter ATP-binding protein | transport | -3.45 | 0.74 | 1.07E-02 |
| I-E, I-F | RS13235 | stress protein | tellurium resistance / response to stress | 1.48 | 0.26 | 3.29E-02 |
| I-E, I-F | RS13240 | TerD, tellurium resistance | tellurium resistance / response to stress | 1.51 | 0.22 | 3.59E-03 |
| I-E, I-F | RS14825 | hypothetical protein | unknown | 3.13 | 0.36 | 1.06E-09 |
| I-E, I-F | RS15240 | RuvB, Holliday junction branch migration DNA helicase | DNA repair/ SOS response | 1.33 | 0.19 | 7.50E-03 |
| I-E, I-F | RS16835 | 6-phospho-beta-glucosidase | carbohydrate metabolism | -1.51 | 0.21 | 2.02E-03 |
| I-E, I-F | RS21055 | RecN, DNA repair protein | DNA repair/ SOS response | 3.19 | 0.23 | 2.24E-25 |
| I-E, I-F | RS21370 | DinI family protein | DNA repair/ SOS response | 2.06 | 0.30 | 1.59E-04 |
| I-E, I-F | RS22350 | LexA, repressor | SOS response | 1.96 | 0.17 | 4.37E-14 |
| I-E | RS05025 | sucrose-6-phosphate hydrolase | sucrose metabolism | 1.29 | 0.19 | 1.71E-02 |
| I-E | RS05275 | iron permease FTR1 | iron transport | -3.04 | 0.52 | 4.70E-04 |

| DE in strains | locus_tag | product | GO molecular function | $\log_2$(FC) | lfcSE | padj |
|---|---|---|---|---|---|---|
| I-E | RS05280 | EfeM/EfeO family lipoprotein | unknown | -2.46 | 0.49 | 1.14E-02 |
| I-E | RS05985 | DNA polymerase II | DNA repair | 1.47 | 0.18 | 1.42E-04 |
| I-E | RS06540 | UvrA, excinuclease ABC subunit | DNA repair / SOS response | 1.41 | 0.21 | 8.94E-03 |
| I-E | RS06820 | LucA, siderophore biosynthesis protein | siderophore biosynthesis | -3.17 | 0.70 | 1.74E-02 |
| I-E | RS06825 | MFS transporter | siderophore biosynthesis | -3.17 | 0.76 | 4.01E-02 |
| I-E | RS06855 | TonB-dependent siderophore receptor | siderophore receptor | -2.76 | 0.63 | 3.82E-02 |
| I-E | RS07775 | hypothetical protein | unknown | 2.07 | 0.42 | 3.29E-02 |
| I-E | RS07980 | RNA-directed DNA polymerase | RNA-dependent DNA replication | -2.22 | 0.44 | 1.73E-02 |
| I-E | RS08430 | ClpB, ATP-dependent chaperone | protein folding / heat shock response | 1.07 | 0.13 | 1.11E-02 |
| I-E | RS08845 | RpoE, RNA polymerase sigma factor | transcription regulation | 1.07 | 0.14 | 3.66E-02 |
| I-E | RS08850 | RseA, anti-sigma-E factor | anti sigma factor | 1.16 | 0.16 | 2.10E-02 |
| I-E | RS10165 | hypothetical protein | unknown | -1.61 | 0.28 | 1.71E-02 |
| I-E | RS10825 | 2,3-dihydroxybenzoate-AMP ligase | siderophore biosynthesis | -3.55 | 0.77 | 1.11E-02 |
| I-E | RS10835 | TonB-dependent siderophore receptor | siderophore receptor | -4.35 | 0.76 | 1.59E-04 |
| I-E | RS10840 | enterochelin esterase | siderophore dependant iron intake | -3.81 | 0.71 | 1.04E-03 |
| I-E | RS10860 | ABC transporter permease | transport | -3.25 | 0.59 | 1.19E-03 |
| I-E | RS10870 | EntS, enterobactin transporter | cellular response to DNA damage stimulus / response to antibiotic | -3.21 | 0.62 | 2.95E-03 |
| I-E | RS10895 | 6-phospho-beta-glucosidase | carbohydrate metabolic process | -1.63 | 0.31 | 4.19E-02 |
| I-E | RS11325 | cytochrome b562 | electron transfer activity | 2.08 | 0.43 | 3.29E-02 |
| I-E | RS12165 | SbnA, 2,3-diaminopropionate biosynthesis protein | diaminopropionate biosynthesis | -1.56 | 0.28 | 3.29E-02 |
| I-E | RS14775 | ferrous iron transport protein A | iron transport | -2.38 | 0.46 | 8.88E-03 |
| I-E | RS15245 | RuvA, Holliday junction branch migration protein | DNA repair/ SOS response | 1.11 | 0.14 | 1.59E-02 |
| I-E | RS16715 | AAA ATPase | unknown | -2.05 | 0.38 | 1.05E-02 |
| I-E | RS16720 | DUF4435 domain-containing protein | unknown | -2.49 | 0.43 | 1.40E-03 |
| I-E | RS16840 | PTS lactose/cellobiose transporter subunit IIA | carbohydrate transport | -1.82 | 0.32 | 9.63E-03 |
| I-E | RS21500 | hypothetical protein | unknown | 2.73 | 0.61 | 3.29E-02 |

## Table S9. Genes differentially expressed in *Serratia* I-F anti-JS26 strain

| DE in strains | locus_tag | product | GO molecular function | $log_2(FC)$ | lfcSE | padj |
|---|---|---|---|---|---|---|
| **Early infection** | | | | | | |
| wt, I-E, I-F | RS02465 | ribose ABC transporter permease | sugar transport | 1.65 | 0.25 | 7.13E-03 |
| wt, I-E, I-F | RS10625 | Ni/Fe-hydrogenase cytochrome b subunit | anaerobic respiration | 3.53 | 0.56 | 8.27E-05 |
| wt, I-E, I-F | RS12610 | NarH, nitrate reductase subunit beta | nitrate reduction | 3.28 | 0.66 | 1.44E-02 |
| wt, I-F | RS05850 | NhaA, Na+/H+ antiporter | sodium transport | 1.46 | 0.22 | 1.56E-02 |
| wt, I-F | RS10630 | cytochrome-c3 hydrogenase | redox process | 3.25 | 0.39 | 1.50E-08 |
| wt, I-F | RS12615 | NarJ, nitrate reductase molybdenum cofactor assembly chaperone | anaerobic respiration | 3.32 | 0.67 | 1.56E-02 |
| I-E, I-F | RS00965 | FadB, fatty acid oxidation complex subunit alpha | fatty acid metabolism | -1.77 | 0.25 | 6.62E-04 |
| I-E, I-F | RS02455 | ribokinase | D-ribose catabolism | 1.93 | 0.14 | 2.26E-17 |
| I-E, I-F | RS02475 | D-ribose pyranase | D-ribose catabolism | 2.85 | 0.56 | 1.62E-02 |
| I-E, I-F | RS10190 | FadL, long-chain fatty acid transporter | fatty acid transport | -2.78 | 0.26 | 1.91E-14 |
| I-F | RS02450 | RbsR, transcriptional regulator | transcriptional regulation | 1.68 | 0.16 | 5.22E-09 |
| I-F | RS03200 | hypothetical protein | unknown | -2.60 | 0.37 | 4.31E-05 |
| I-F | RS04170 | GlpC, anaerobic glycerol-3-phosphate dehydrogenase subunit C | anaerobic respiration | 2.37 | 0.35 | 2.28E-04 |
| I-F | RS10635 | HyaD/HybD family hydrogenase maturation endopeptidase | protein processing | 2.60 | 0.38 | 6.98E-05 |
| I-F | RS10885 | PRD domain-containing protein | transcription regulation | -2.63 | 0.44 | 1.68E-03 |
| I-F | RS16980 | SpeF, ornithine decarboxylase | spermidine biosynthesis | 3.24 | 0.61 | 5.32E-03 |
| **Mid-infection** | | | | | | |
| wt, I-E, I-F | RS05850 | NhaA, Na+/H+ antiporter | sodium transport | 2.14 | 0.28 | 1.73E-05 |
| wt, I-E, I-F | RS05855 | NhaR, transcriptional activator | sodium transport | 1.46 | 0.18 | 3.43E-04 |
| wt, I-E, I-F | RS07090 | DUF2502 domain-containing protein | unknown | 2.24 | 0.32 | 1.52E-04 |
| wt, I-E, I-F | RS10685 | DUF2002 domain-containing protein | unknown | 3.29 | 0.44 | 1.12E-06 |
| wt, I-E, I-F | RS18615 | ABC transporter substrate-binding protein | transport | -4.24 | 0.96 | 3.15E-02 |
| wt, I-E, I-F | RS18685 | MenF, isochorismate synthase | menaquinone biosynthesis | 1.73 | 0.16 | 8.27E-10 |
| wt, I-F | RS11860 | glycine zipper 2TM domain-containing protein | unknown | 2.94 | 0.56 | 8.06E-03 |
| I-E, I-F | RS07645 | DNA polymerase IV | DNA repair / SOS response | 1.97 | 0.26 | 3.34E-05 |

| DE in strains | locus_tag | product | GO molecular function | $log_2(FC)$ | lfcSE | padj |
|---|---|---|---|---|---|---|
| I-E, I-F | RS08295 | RecA, recombinase | DNA repair / SOS response | 2.25 | 0.38 | 3.11E-03 |
| I-E, I-F | RS08300 | RecX, recombination regulator | DNA repair / SOS response | 1.91 | 0.30 | 2.68E-03 |
| I-E, I-F | RS08850 | RseA, anti-sigma-E factor | anti-sigma binding | 1.31 | 0.19 | 3.50E-02 |
| I-E, I-F | RS09495 | SulA, cell division inhibitor | cellular response to DNA damage stimulus / SOS response | 2.55 | 0.28 | 1.81E-09 |
| I-E, I-F | RS14825 | hypothetical protein | unknown | 2.56 | 0.49 | 1.31E-02 |
| I-E, I-F | RS21055 | RecN, DNA repair protein | DNA repair/ SOS response | 3.06 | 0.26 | 2.47E-18 |
| I-E, I-F | RS21370 | DinI family protein | DNA repair/ SOS response | 1.78 | 0.25 | 7.05E-04 |
| I-E, I-F | RS22350 | LexA, repressor | DNA repair / SOS response | 1.70 | 0.27 | 8.06E-03 |
| I-F | RS00630 | RNA-binding S4 domain protein | response to heat | 1.70 | 0.28 | 1.31E-02 |
| I-F | RS03060 | IbpA, heat shock protein | heat shock response | 2.04 | 0.29 | 2.23E-04 |
| I-F | RS03065 | IbpB, heat shock chaperone | heat shock response | 1.85 | 0.29 | 3.31E-03 |
| I-F | RS09530 | HspQ, heat shock protein | heat shock response | 2.45 | 0.37 | 1.93E-04 |
| I-F | RS22385 | FxsA, cytoplasmic membrane protein | unknown | 1.81 | 0.34 | 4.96E-02 |
| **Late infection** | | | | | | |
| wt, I-E, I-F | RS05850 | NhaA, Na+/H+ antiporter | sodium transport | 1.74 | 0.17 | 1.22E-09 |
| wt, I-E, I-F | RS07090 | DUF2502 domain-containing protein | unknown | 2.92 | 0.38 | 3.19E-07 |
| wt, I-E, I-F | RS07105 | DUF1107 domain-containing protein | unknown | 2.60 | 0.41 | 2.10E-04 |
| wt, I-E, I-F | RS09545 | transmembrane protein | negative regulation of apoptotic process | 1.71 | 0.26 | 2.50E-03 |
| wt, I-E, I-F | RS09860 | Mannose-1-phosphate guanylyltransferase/mannose-6-phosphate isomerase | polysaccharide biosynthetic process | 1.40 | 0.21 | 1.03E-02 |
| wt, I-E, I-F | RS10685 | DUF2002 domain-containing protein | unknown | 4.80 | 0.48 | 2.61E-15 |
| wt, I-E, I-F | RS10690 | hypothetical protein | unknown | 4.49 | 1.02 | 1.40E-02 |
| wt, I-E, I-F | RS11860 | glycine zipper 2TM domain-containing protein | unknown | 3.92 | 0.33 | 2.61E-21 |
| wt, I-E, I-F | RS12140 | 6-phospho-beta-glucosidase | carbohydrate metabolism | -3.44 | 0.79 | 2.50E-02 |
| wt, I-E, I-F | RS12150 | esterase | carbohydrate metabolism | -3.77 | 0.85 | 1.78E-02 |
| wt, I-E, I-F | RS12155 | hypothetical protein | unknown | -3.77 | 0.87 | 2.25E-02 |
| wt, I-E, I-F | RS12340 | OsmB, osmotically-inducible lipoprotein | osmotic stress | 2.22 | 0.47 | 4.18E-02 |
| wt, I-E, I-F | RS15300 | CutC, copper homeostasis protein | copper homeostasis | 1.48 | 0.23 | 1.34E-02 |

| DE in strains | locus_tag | product | GO molecular function | $log_2$(FC) | lfcSE | padj |
|---|---|---|---|---|---|---|
| wt, I-E, I-F | RS15785 | DmsD, Tat proofreading chaperone | protein folding | 2.26 | 0.35 | 3.10E-04 |
| wt, I-E, I-F | RS16005 | serine hydrolase | proteolysis | 1.97 | 0.37 | 1.82E-02 |
| wt, I-E, I-F | RS16385 | hypothetical protein | unknown | -2.21 | 0.42 | 1.24E-02 |
| wt, I-E, I-F | RS18680 | MenD, 2-succinyl-5-enolpyruvyl-6-hydroxy-3-cyclohexene-1-carboxylic-acid synthase | menaquinone biosynthesis | 1.61 | 0.19 | 9.01E-06 |
| wt, I-E, I-F | RS18685 | MenF, isochorismate synthase | menaquinone biosynthesis | 1.96 | 0.12 | 1.79E-25 |
| wt, I-E, I-F | RS22235 | hypothetical protein | unknown | 2.03 | 0.35 | 6.04E-03 |
| wt, I-E, I-F | RS22565 | OsmY, molecular chaperone | protein folding / Response to osmotic stress | 2.06 | 0.38 | 1.29E-02 |
| wt, I-F | RS03770 | PutA, trifunctional transcriptional regulator/proline dehydrogenase/L-glutamate gamma-semialdehyde dehydrogenase | proline biosynthetic process/ transcriptional regulation | 2.81 | 0.62 | 2.56E-02 |
| wt, I-F | RS05855 | NhaR, transcriptional activator | sodium transport | 1.14 | 0.16 | 3.05E-02 |
| wt, I-F | RS10950 | hypothetical protein | unknown | 2.93 | 0.53 | 1.58E-03 |
| wt, I-F | RS10955 | sugar ABC transporter permease | sugar transport | 2.71 | 0.53 | 8.00E-03 |
| wt, I-F | RS12185 | non-ribosomal peptide synthetase | catalytic activity | -1.94 | 0.31 | 2.27E-03 |
| wt, I-F | RS18615 | ABC transporter substrate-binding protein | transport | -4.10 | 0.94 | 1.82E-02 |
| wt, I-F | RS18670 | MenB, 1,2C4-dihydroxy-2-naphthoyl-CoA synthase | menaquinone biosynthesis | 1.56 | 0.26 | 1.40E-02 |
| wt, I-F | RS18675 | MenH, 2-succinyl-6-hydroxy-2%2C4-cyclohexadiene-1-carboxylate synthase | menaquinone biosynthesis | 1.81 | 0.28 | 2.73E-03 |
| I-E, I-F | RS01685 | oligopeptidase A | proteolysis | 1.53 | 0.25 | 1.45E-02 |
| I-E, I-F | RS03060 | IbpA, heat shock protein | heat shock response | 1.75 | 0.23 | 9.21E-05 |
| I-E, I-F | RS04105 | UvrD, DNA helicase II | DNA repair | 1.20 | 0.14 | 1.30E-03 |
| I-E, I-F | RS07645 | DinB, DNA polymerase IV | DNA repair / SOS response | 1.91 | 0.17 | 2.06E-12 |
| I-E, I-F | RS08295 | RecA, recombinase | DNA repair / SOS response | 2.32 | 0.23 | 6.28E-11 |
| I-E, I-F | RS08300 | RecX, recombination regulator | DNA repair / SOS response | 2.03 | 0.17 | 2.26E-14 |
| I-E, I-F | RS09495 | SulA, cell division inhibitor | SOS response | 2.36 | 0.18 | 7.71E-19 |
| I-E, I-F | RS09530 | HspQ, heat shock protein | Heat shock response | 2.08 | 0.24 | 7.63E-08 |
| I-E, I-F | RS10830 | isochorismate synthase | siderophore biosynthesis | -3.82 | 0.83 | 1.24E-02 |
| I-E, I-F | RS10845 | MbtH family protein | siderophore biosynthesis | -3.75 | 0.92 | 4.67E-02 |
| I-E, I-F | RS10855 | ABC transporter ATP-binding protein | transport | -3.80 | 0.92 | 3.88E-02 |
| I-E, I-F | RS13235 | stress protein | tellurium resistance / response to stress | 1.87 | 0.31 | 4.60E-03 |

| DE in strains | locus_tag | product | GO molecular function | $log_2$(FC) | lfcSE | padj |
|---|---|---|---|---|---|---|
| I-E, I-F | RS13240 | Tellurium resistance | tellurium resistance / response to stress | 1.73 | 0.30 | 1.54E-02 |
| I-E, I-F | RS14825 | hypothetical protein | unknown | 2.56 | 0.39 | 1.09E-04 |
| I-E, I-F | RS15240 | RuvB, Holliday junction branch migration DNA helicase | DNA repair/ SOS response | 1.12 | 0.14 | 1.34E-02 |
| I-E, I-F | RS16835 | 6-phospho-beta-glucosidase | carbohydrate metabolic process | -1.21 | 0.15 | 3.21E-03 |
| I-E, I-F | RS21055 | RecN, DNA repair protein | DNA repair/ SOS response | 2.95 | 0.19 | 5.09E-34 |
| I-E, I-F | RS21370 | DinI family protein | DNA repair/ SOS response | 1.75 | 0.17 | 4.05E-09 |
| I-E, I-F | RS22350 | LexA, repressor | SOS response | 1.63 | 0.15 | 1.59E-09 |
| I-F | RS00620 | IgaA, intracellular growth attenuator family protein | signal transduction / stress response | 1.25 | 0.18 | 2.50E-02 |
| I-F | RS00630 | RNA-binding S4 domain protein | cellular response to heat | 1.50 | 0.24 | 1.40E-02 |
| I-F | RS01030 | RmuC, DNA recombination protein | DNA recombination | 1.96 | 0.29 | 3.22E-04 |
| I-F | RS01690 | RsmJ, 16S rRNA (guanine(1516)-N(2))-methyltransferase | rRNA methylation | 1.36 | 0.22 | 3.80E-02 |
| I-F | RS03065 | IbpB, heat shock chaperone | heat shock response | 1.65 | 0.24 | 1.34E-03 |
| I-F | RS06570 | Cas5f, type I-F CRISPR-associated protein | CRISPR type I-F immunity | 1.74 | 0.34 | 4.88E-02 |
| I-F | RS06575 | Cas8f, type I-F CRISPR-associated protein | CRISPR type I-F immunity | 1.84 | 0.31 | 7.98E-03 |
| I-F | RS06580 | Cas3, type I-F CRISPR-associated helicase | CRISPR type I-F immunity | 2.03 | 0.26 | 6.03E-06 |
| I-F | RS06585 | Cas1, type I-F CRISPR-associated endonuclease | CRISPR type I-F immunity | 2.19 | 0.29 | 1.20E-05 |
| I-F | RS12180 | cyclic peptide export ABC transporter | transport | -1.91 | 0.33 | 9.01E-03 |
| I-F | RS13245 | TerB, tellurite resistance | tellurium resistance | 1.72 | 0.31 | 2.52E-02 |
| I-F | RS22385 | FxsA cytoplasmic membrane protein | unknown | 1.41 | 0.21 | 9.86E-03 |

## References


1. Jackson SA, Birkholz N, Malone LM, Fineran PC. 2019. Imprecise Spacer Acquisition Generates CRISPR-Cas Immune Diversity through Primed Adaptation. Cell Host Microbe 25:250-260.
2. Patterson AG, Jackson SA, Taylor C, Evans GB, Salmond GPC, Przybilski R, Staals RHJ, Fineran PC. 2016. Quorum Sensing Controls Adaptive Immunity through the Regulation of Multiple CRISPR-Cas Systems. Mol Cell 64:1102-1108.
3. Smith LM, Jackson SA, Malone LM, Ussher JE, Gardner PP, Fineran PC. 2021. The Rcs stress response inversely controls surface and CRISPR-Cas adaptive immunity to discriminate plasmids and phages. Nat Microbiol doi:10.1038/s41564-020-00822-7.
4. Thoma S, Schobert M. 2009. An improved *Escherichia coli* donor strain for diparental mating. FEMS Microbiol Lett 294:127-32.
5. Thomson NR, Crow MA, McGowan SJ, Cox A, Salmond GPC. 2000. Biosynthesis of carbapenem antibiotic and prodigiosin pigment in *Serratia* is under quorum sensing control. Molecular Microbiology 36:539-556.
6. Hampton HG, McNeil MB, Paterson TJ, Ney B, Williamson NR, Easingwood RA, Bostina M, Salmond GP, Fineran PC. 2016. CRISPR-Cas gene-editing reveals RsmA and RsmC act through FlhDC to repress the SdhE flavinylation factor and control motility and prodigiosin production in *Serratia*. Microbiology 162:1047-58.
